# Chilling Affects Phytohormone and Post-Embryonic Development Pathways during Bud Break and Fruit Set in Apple (*Malus domestica* Borkh.)

**DOI:** 10.1038/srep42593

**Published:** 2017-02-15

**Authors:** Gulshan Kumar, Khushboo Gupta, Shivalika Pathania, Mohit Kumar Swarnkar, Usha Kumari Rattan, Gagandeep Singh, Ram Kumar Sharma, Anil Kumar Singh

**Affiliations:** 1 Department of Biotechnology, CSIR-Institute of Himalayan Bioresource Technology, Palampur, 176061, India; 2 Academy of Scientific and Innovative Research, New Delhi, India.; 3 ICAR-Indian Institute of Agricultural Biotechnology, PDU Campus, IINRG, Namkum, Ranchi-834010 (JH), India.

## Abstract

The availability of sufficient chilling during bud dormancy plays an important role in the subsequent yield and quality of apple fruit, whereas, insufficient chilling availability negatively impacts the apple production. The transcriptome profiling during bud dormancy release and initial fruit set under low and high chill conditions was performed using RNA-seq. The comparative high number of differentially expressed genes during bud break and fruit set under high chill condition indicates that chilling availability was associated with transcriptional reorganization. The comparative analysis reveals the differential expression of genes involved in phytohormone metabolism, particularly for Abscisic acid, gibberellic acid, ethylene, auxin and cytokinin. The expression of *Dormancy Associated MADS*-box, *Flowering Locus C*-like, *Flowering Locus T*-like and *Terminal Flower 1*-like genes was found to be modulated under differential chilling. The co-expression network analysis indentified two high chill specific modules that were found to be enriched for “post-embryonic development” GO terms. The network analysis also identified hub genes including *Early flowering 7, RAF10, ZEP4* and *F-box*, which may be involved in regulating chilling-mediated dormancy release and fruit set. The results of transcriptome and co-expression network analysis indicate that chilling availability majorly regulates phytohormone-related pathways and post-embryonic development during bud break.

Temperature plays a crucial role in determining the phenological events of perennial plants. In the changing climate scenario, the productivity and distribution of perennial plants is affected by the phenological traits of the vegetative and reproductive buds. The involvement of molecular mechanism analogous to vernalization process in Arabidopsis, has been proposed for chilling-dependent bud dormancy release in perennial species^1^. In Arabidopsis, the expression of *FLOWERING LOCUS T (FT*) is negatively regulated by *FLOWERING LOCUS C (FLC*)^2^. Extended cold exposure makes the plant competent to flower by suppressing the expression of *FLC* through epigenetic regulation. Bud break in poplar has been found to be associated with expression of *PtFT1*, an ortholog of Arabidopsis flowering promoter *FT*^3^. Although, the presence of *FLC*-like genes has been reported in few perennial species, their expression level does not coincide with the dormancy release^4^,^5^. Analysis of *evergrowing* peach mutant, which was unable to enter dormancy led to identification of *DORMANCY ASSOCIATED MADS-BOX (DAM*) gene^6^. The expression and associated epigenetic modifications during dormancy release, and over-expression studies have identified *DAM* as potential flower repressor in perennial plants^7^,^8^,^9^. Recently, the expression of *DAM* genes was not found to be coinciding with dormancy release in apple, however a possible role of *FLC*-like genes was proposed in dormancy regulation^10^.

In addition, role of various plant growth regulators during bud dormancy establishment and its release has been evaluated in several studies^11^. In apple buds, the level of endogenous ABA was reported to be higher during endodormancy, thereafter it was found to be decreased with dormancy release^12^. The inhibitory effect of ABA on axillary buds of apple was also reported^13^. Exogenous application of GA was found to substitute the chilling requirement with up-regulation of β-1,3-glucanase, which helps in breakdown of a cell wall component, callose during dormancy release in poplar^14^. In grape buds, chemical induced bud dormancy was reported to be associated with regulation of *ethylene response factors (ERF*), which resulted in temporary induction of endogenous ethylene synthesis^15^. Similarly, increased ethylene level during dormancy induction in birch (*Betula pendula*) was also reported^16^. It was also reported that ethylene production in buds may lead to accumulation of ABA, and this observation was in accordance with studies on potato tubers^17^. Auxin is another hormone, which is known to be involved in dormancy induction and maintenance. Higher level of auxin during dormant period and its dynamic relation with apical and lateral bud dominance has been shown^18^. A recent study using microarray suggested the possible role of auxin during dormancy release in apple^10^.

Apple (*Malus domestica* Borkh) is one of the most widely cultivated fruit trees in the temperate zones of the world. As winter approaches, already developed paradormant floral buds gradually switch to endodormant state and remain dormant until they accumulate sufficient chilling units (CUs; temperature ≤7 °C for one h) during winter. Even after the accumulation of sufficient CUs, persistent unfavorable environmental conditions gradually shift floral buds from endodormant to ecodormant state. Rise in temperature makes the ecodormant buds committed to re-initiate their growth. Change in climatic conditions of North-Western Himalayas led to decreasing trend of low temperature during November to February months^19^. This led to decrease in available CUs necessary for dormancy release in apple and other fruit trees^20^. Lack of sufficient CUs delays the onset of flowering and also causes irregular breaking of bud dormancy, which eventually affects the fruit yield and quality. While, accumulation of sufficient CUs during winter has significant positive correlation with quality and productivity of apple fruit^21^. Thus, comprehensive understanding of underlying molecular mechanism of dormancy release is prerequisite to encounter the challenges of changing climatic conditions. So far, only two studies related to transcriptome profiling during bud beak in apple have been reported^10^,^22^. However, the effect of differential chilling availability on transcriptional profile across the bud dormancy stages has not been investigated, yet. Therefore, the present study was aimed to identify the differentially expressed genes (DEGs) associated with bud dormancy and fruit set under differential chilling availability.

## Results

### RNA-seq data assembly and annotation

**Figure 1:**
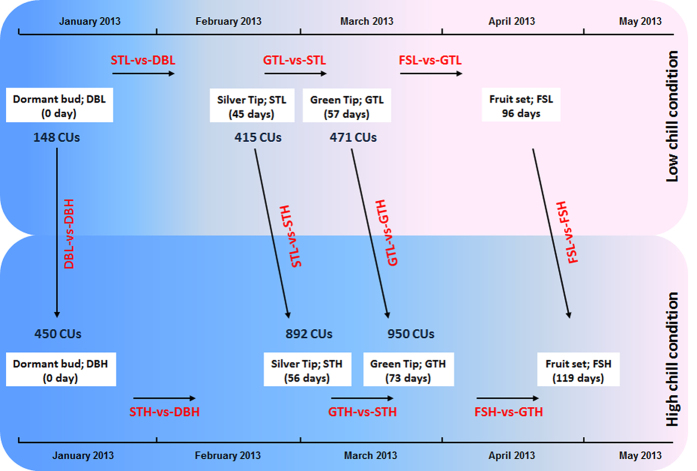
Sample abbreviations, their collection intervals, comparative conditions and accumulated chilling units (CUs) during winter dormancy. The developmental stages in sequential phenological events were compared in six comparative conditions *viz:* dormant bud and silver tip (STL-vs-DBL; STH-vs-DBH), silver tip and green tip (GTL-vs-STL; GTH-vs-STH) and green tip and initial fruit set (FSL-vs-GTL; FSH-vs-GTH), under respective low (L) and high (H) chill conditions. The remaining four comparative conditions include comparison of similar developmental stage under differential chilling conditions *viz:* dormant bud (DBL-vs-DBH), silver tip (STL-vs-STH), green tip (GTL-vs-GTH) and fruit set (FSL-vs-FSH), considering high chill as control condition. The Utah model was used to calculate the accumulated CUs. The accumulated CUs under both locations indicate that apple buds accumulate more CUs under high chill condition as compared to low chill condition. The high chill and low chill conditions are represented by Palchan and Seobag, respectively.

The RNA-seq analysis of four developmental stages *viz*. dormant bud (DB), silver tip (ST), green tip (GT) and initial fruit set (FS) of apple under differential chilling conditions was performed. The sample abbreviations, accumulated chilling units (CUs) and schematic representation of comparative conditions are given in Fig. 1. The pictures of morphological state of collected samples are given in Supplementary Fig. S1. The sequencing of RNA-seq libraries of eight samples generated 97,960,812 raw reads. The quality filtering led to removal of 7,080,502 low quality reads, yielding 90,880,310 high quality reads. Since, only the draft genome of apple is available, the *de novo* assembly of 90,880,310 high quality reads was performed that yielded 68,455 contigs, with N50 of 766 bp and an average length of 603 bp. Out of 68,455 contigs, 57,603 (84.1%) were successfully annotated against non-redundant, NCBI protein database using BLASTX program.

### Comparative analysis of DEGs under differential chilling conditions

**Figure 2:**
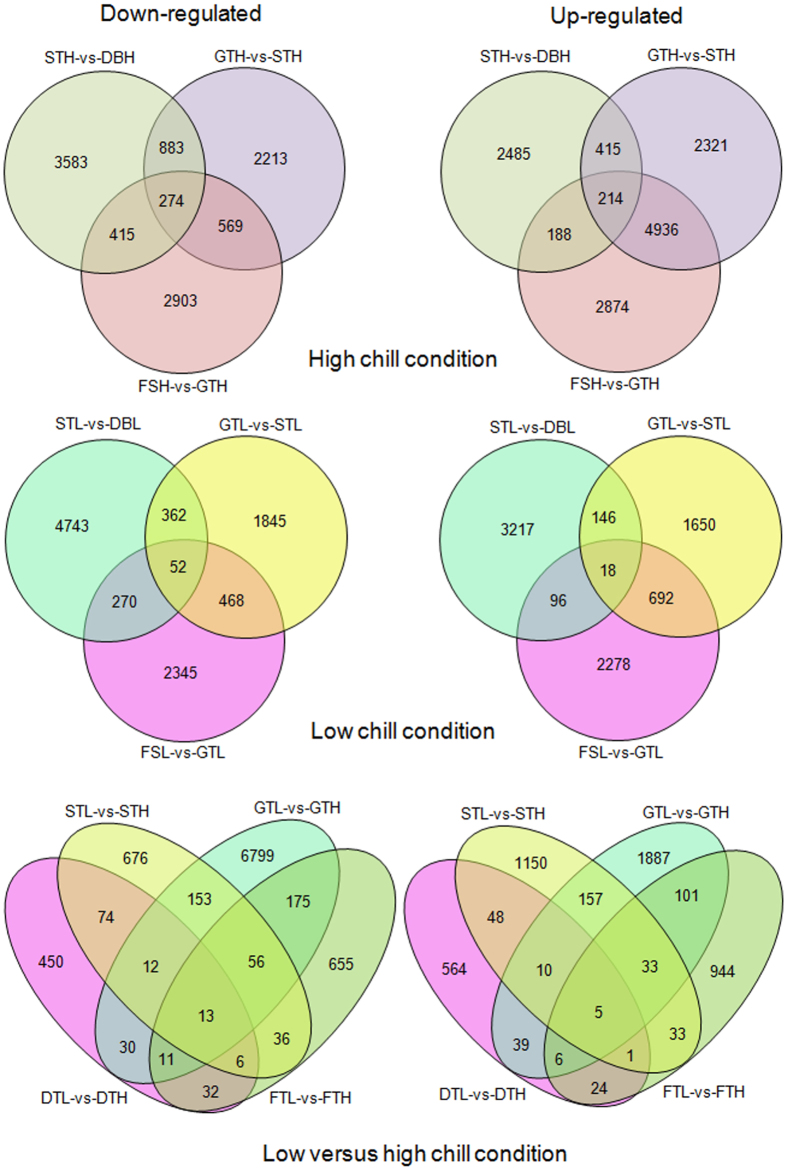
The Venn diagrams showing common differentially expressed transcripts. The number of transcripts common in two or more comparative conditions are enclosed in overlapping portion of the circles.

In the present analysis, DEGs were identified in ten comparative conditions (Fig. 1). The summary of DEGs in corresponding conditions of different locations is given in Supplementary Table S1. Transition from dormant to active growth (GTH-vs-STH) and, initial fruit set stage (FSH-vs-GTH) under high chill conditions was observed to be associated with higher number of DEGs as compared to low chill conditions. From the total DEGs of GTH-vs-STH and FSH-vs-GTH, higher number of contigs was found to be upregulated. Similarly, GTL-vs-GTH was also found to have higher number of DEGs, however, FSL-vs-FSH was found to have low number of DEGs. Interestingly, the higher number of downregulated DEGs in GTL-vs-GTH, and higher number of upregulated DEGs in GTH-vs-STH and FSH-vs-GTH comparison indicates that chilling availability is determinant in regulating the expression of genes during dormancy release in apple (Fig. 2). Therefore, results indicate that major transcriptional reorganization occurred during initiation of active growth (GT) and the subsequent fruit set (FS) in apple.

### GO and KEGG enrichment analysis of DEGs

In the present investigation, irrespective of chilling conditions, the GO terms such as response to stimulus and response to stress were highly enriched in STL-vs-DBL and STH-vs-DBH comparisons (Supplementary Table S2). In GTL-vs-STL, the cellular component biogenesis and photosynthesis were found to be highly enriched, while cellular metabolic process was found to be enriched in GTH-vs-STH (Supplementary Table S2). During fruit set, post-embryonic development was found to be enriched in both FSL-vs-GTL and FSH-vs-GTH. In case of DBL-vs-DBH and STL-vs-STH, the oxidation reduction, post-embryonic development and lipid localization were among the highly enriched GO terms. While, in GTL-vs-GTH, response to heat and DNA replication were among the highly enriched GO terms. Whereas, post-embryonic development and generation of precursor metabolites and energy were the highly enriched GO terms in FSL-vs-FSH (Supplementary Table S2). From the GO enrichment analysis of DEGs under low and high chill conditions, it was observed that dormancy release under high chill was mostly associated with the post-embryonic development and lipid metabolism, while it was mostly associated with GO terms, such as response to stimulus and response to stress under low chill conditions.

The DEGs of different comparative conditions were also subjected to KEGG enrichment analysis using KOBAS. In STL-vs-DBL, the enrichment of fatty acid metabolism, plant hormone signal transduction and circadian rhythm pathways was observed (Supplementary Table S3). While, in STH-vs-DBH, cell cycle, arginine and proline metabolism, AMPK signaling pathway and regulation of actin cytoskeleton were found to be enriched. In GTL-vs-STL, the enrichment of important pathways such as, plant hormone signal transduction, starch and sucrose metabolism, and porphyrin and chlorophyll metabolism was observed. Whereas, in GTH-vs-STH, phenylpropanoid biosynthesis, VEGF signaling pathway and diterpenoid biosynthesis pathways were the important enriched pathways. In FSL-vs-GTL, enrichment of pathways such as ribosome biogenesis, starch and sucrose metabolism, and cysteine and methionine metabolism was observed. While, in FSH-vs-GTH, the enrichment of fructose and mannose metabolism, glycerolipid metabolism, Ras signaling pathway and mTOR signaling pathway was observed. In case of comparison of similar stages under differential chilling conditions, the enrichment of phenylpropanoid biosynthesis in STL-vs-STH, ether lipid metabolism in GTL-vs-GTH and oxidative phosphorylation in FSL-vs-FSH was observed among the down-regulated DEGs (Supplementary Table S3). These observations revealed that bud break and fruit set under high chill condition were mostly associated with the signaling pathways and lipid metabolism, while it was mostly associated with carbon metabolism-related pathways under low chill conditions.

### Phytohormone related pathways

**Figure 3:**
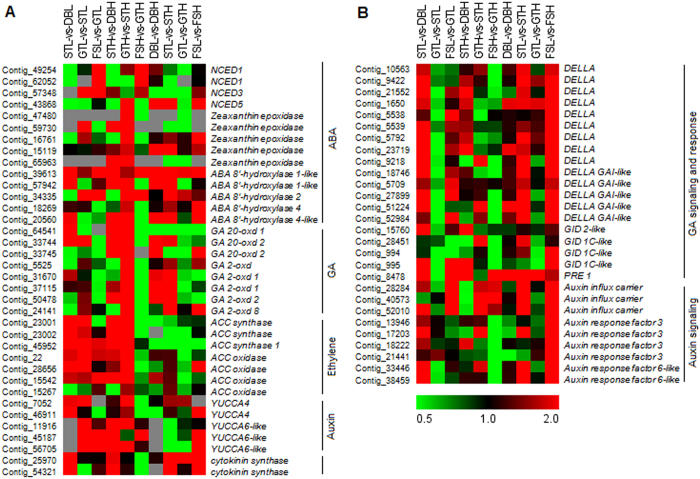
The heat map representation of genes involved in phytohormone metabolism and signaling. Heat map showing relative expression of genes involved in ABA, GA, ethylene, Auxin and cytokinin metabolism (**A**) and, GA and Auxin signaling (**B**). The expression values are RNA-seq FPKM values. The grey color in the heat map represents no expression.

The GO term ‘response to stress’ was found to be enriched in DEGs of dormant bud stages (Supplementary Table S2). Since ABA is associated with stress response, carotenoid metabolic pathway was analyzed for ABA metabolism. Expression of *NCED3* (Contig_57348), a key gene involved in ABA biosynthesis, was found to be increased along the bud break and fruit set under high chill conditions (Fig. 3A). In contrast, transcripts of *NCED1* (Contig_49254 and Contig_62052) were found to be down-regulated in STL-vs-DBL and STH-vs-DBH before bud break, while upregulation of these transcripts was observed during fruit set in FSL-vs-GTL and FSH-vs-GTH. Almost similar expression pattern was observed for *NCED5* (Contig_43868), however, up-regulation of this transcript was observed in GTH-vs-STH. In comparison of similar phenological stages under differential chilling conditions, the expression of *NCED3* was observed to be downregulated during bud break under low chill conditions. In addition, the high expression of *ABA 8*′*-hydroxylase*, involved in ABA degradation, was observed under low chill conditions in comparison to high chill conditions (Fig. 3A).

Three transcripts of *gibberellin 20-oxidase (GA 20-oxd*; Contig_33744, Contig_33745 and Contig_64541), a key gene in gibberellic acid (GA) biosynthesis, were found to be up-regulated in STH-vs-DBH and GTH-vs-STH, while only one transcript (Contig_33744) was found to be up-regulated in STL-vs-DBL and GTL-vs-STL (Fig. 3A). Similarly, under differential chilling conditions, the expression of *GA 20-oxd* transcripts was found to be high in DBL-vs-DBH, thereafter decreased in the STL-vs-STH, GTL-vs-GTH and FSL-vs-FSH. Five transcripts (Contig_5525, Contig_24141, Contig_31670, Contig_37115 and Contig_50478) of *gibberellin 2-oxidase (GA 2-oxd*) were found to have higher expression in GTH-vs-STH and GTL-vs-STL, where fold change was found to be more in GTH-vs-STH. In addition, these transcripts were found to be up-regulated in STL-vs-STH, while in GTL-vs-GTH and FSL-vs-FSH, their expression was either down-regulated or unaltered under low chill condition. Moreover, majority of the *DELLA* transcripts were found to have higher expression in STL-vs-STH and FSL-vs-FSH, while their expression was observed to be either unaltered or down-regulated in GTL-vs-GTH (Fig. 3B).

Three *ACC synthase (ACS*) transcripts (Contig_23001, Contig_23003 and Contig_45952), associated with ethylene biosynthesis, were found to be gradually up-regulated along the bud break and thereafter decreased during subsequent fruit set. While, expression of these transcripts was found to be higher in GTH-vs-STH and STL-vs-DBL (Fig. 3A). The relative expression of *ACS* transcripts in all the comparisons of similar stages under differential chilling conditions was observed to be more under high chill conditions. In contrast, the *ACC oxidase (ACO*) transcript (Contig_15267), which oxidizes ACC to ethylene, was found to have down-regulated expression in STH-vs-DBH and STL-vs-DBL. However, slight increase in its expression was observed in GTH-vs-STH and FSL-vs-GTL. In similar stages under differential chilling conditions, DBL-vs-DBH and GTL-vs-GTH were observed to have low relative expression of *ACO*, while its expression was high in STL-vs-STH (Fig. 3A). Therefore, results indicate that high chill conditions might be associated with more ethylene biosynthesis.

Similar to ABA, the GO term ‘Response to Auxin stimulus’ and ‘Auxin polar transport’ was also found to be enriched in DEGs of few (GTL-vs-GTH and FSL-vs-GTL) among the other comparative conditions, therefore, the expression of *YUCCA* genes, which catalyze rate-limiting step of auxin biosynthesis, was analyzed (Supplementary Fig. S2). The expression of three transcripts related to *YUCCA6*-like genes (Contig_11916, Contig_45187 and Contig_56705), was found to be gradually increased along the bud break with higher expression under high chill condition (Fig. 3A). The similar expression of *YUCCA4* transcripts (Contig_7052 and Contig_46911) was observed, however with maximum expression during GT stage. Thereafter, its expression was found to be either low or undetectable in fruit set stage (Fig. 3A). Among the auxin signaling genes, the expression of *auxin influx carrier* (Contig_28284, Contig_40573 and Contig_52010), *auxin response factor3* (Contig_13946, Contig_17203, Contig_18222 and Contig_21441) and *auxin response factor6*-like (Contig_33446 and Contig_38459) was found to be upregulated under low chill conditions (Fig. 3B). These results suggest that auxin metabolism is modulated by temperature, where low chill condition is associated with higher expression of auxin biosynthesis genes as well as genes involved in auxin response during silver tip and fruit set stages as compared to high chill conditions. A gene encoding *cytokinin synthase* (Contig_25970 and Contig_54321), involved in cytokinin biosynthesis, was found to have upregulated expression in STH-vs-DBH and STL-vs-DBL, with more upregulation under low chill conditions (Fig. 3A). These results suggested that, low chill condition was associated with high cytokinin biosynthesis.

### Analysis of genes involved in regulating dormancy release and flowering time

**Figure 4:**
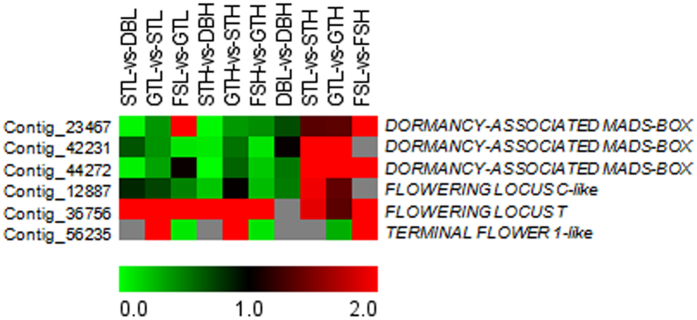
The heat map representation of genes involved in flowering time regulation. Heat map showing relative expression of genes involved in dormancy release and flowering time regulation. The expression values are RNA-seq FPKM values. The grey color in the heat map represents no expression.

Three MADS-box gene transcripts (Contig_23467, Contig_42231 and Contig_44272) showed high similarity with previously identified *DORMANCY ASSOCIATED MADS-BOX (DAM*) genes. All these transcripts were found to have decreased expression along with bud break under both chilling conditions, however the downregulation was more in STH-vs-DBH as compared to STL-vs-DBL (Fig. 4). The analysis of similar developmental stages under differential chilling conditions showed that these transcripts have low expression in DBL-vs-DBH, while higher expression was observed in STL-vs-STH, GTL-vs-GTH and FSL-vs-FSH. The transcript (Contig_12887) that showed similarity with pear *FLC*-like gene, was found to have maximum expression during dormant bud stage and thereafter downregulated in bud break and fruit set stages. However, decrease in expression towards the bud break was more rapid (0.2 fold in STH-vs-DBH) under high chill condition as compared to low chill condition (0.85 fold in STL-vs-DBL) (Fig. 4). Moreover, basic expression level of *FLC*-like gene was found to be higher during the bud break under low chill conditions. The expression of *FT*-like (Contig_36756), a gene belonging to PEBP family, was found to be gradually increased towards the bud break and fruit set, where higher expression of *FT*-like gene was observed under low chill conditions. The *TERMINAL FLOWER 1 (TFL1*)-like (Contig_56235), a member of PEBP family associated with repression of precocious flowering, was found to be expressed only in actively growing buds (GT) with downregulated expression in GTL-vs-GTH (Fig. 4).

### Expression of genes involved in epigenetic regulation

**Figure 5:**
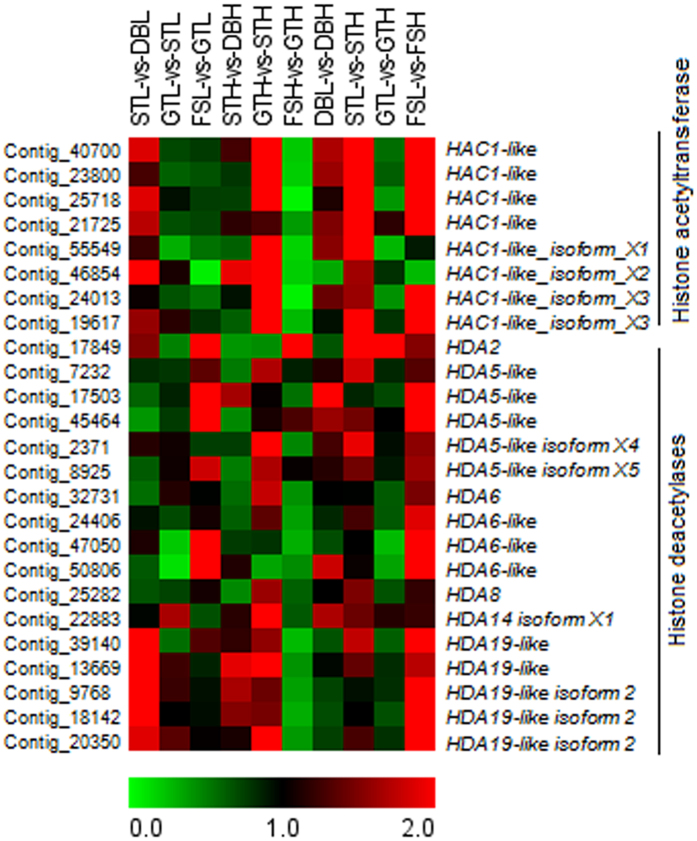
The heat map representation of genes involved in histone modifications. Heat map showing relative expression of genes involved in histone acetylation and deacetylation. The expression values are RNA-seq FPKM values. The grey color in the heat map represents no expression.

In the present analysis, majority of *Histone acetyltransferase1 (HAC1*)-like transcripts were found to be upregulated towards the initiation of active growth under high chill conditions in GTH-vs- STH (Fig. 5). However, the basic transcript levels of *HAC1*-like transcripts were found to be higher under low chill conditions. The expression of histone deacetylase (*HDA*), involved in transcriptional repression, including *HDA14* and *HDA19* was found to be either upregulated or unaltered during initiation of active growth under high chill condition in GTH-vs-STH. In contrast, the expression of *HDA2, HDA5, HDA6* and *HDA8* was found to be either downregulated or unaltered during dormancy release towards the fruit set through initiation of active growth, however, either upregulated or unaltered expression was observed in GTH-vs-STH and FSL-vs-GTL (Fig. 5).

### Quantitative real-time PCR (qRT-PCR) validation of FPKM based expression values

In order to validate the FPKM-based expression values, qRT-PCR was carried out for selected genes. The fourteen selected genes were related to floral time regulation (*DAMs, FLC*-like, *TFL*-like) and phytohormone metabolism (ABA: *NCED1* and *NCED5*; GA: *GA 20-oxd* and *GA 2-oxd*; ethylene: *ACO* and *ACS*; auxin: *YUCCA4*; cytokinin: *cytokinin synthase*). The sequence of the contigs selected for qRT–PCR analysis and their respective primers are given in Supplementary Table S4. The expression pattern of *DAMs, FLC*-like, *TFL*-like, *NCED1, NCED5, GA 20-oxd, GA 2-oxd, ACO, ACS, YUCCA4* and *cytokinin synthase*, observed through the FPKM and qRT-PCR based expression values were moderately correlated with each other, whereas few samples show obscure correlation in FPKM and qRT-PCR expression values (Supplementary Fig. S3). The Pearson correlation coefficient between fold change FPKM and qRT-PCR for the floral time regulation genes was 0.599 (p-value = 2.16e–06), while it was 0.508 (p-value = 3.0e–08) for genes related to phytohormone metabolism.

### Network construction and enrichment analysis

**Figure 6:**
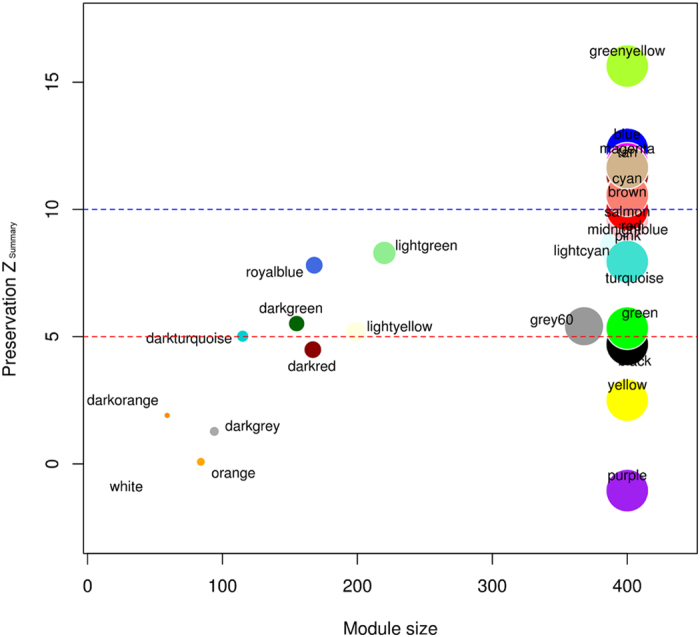
The preservation statistics for high chill modules. The modules with Z_summary_ statistics below three, were considered as non-conserved modules.

For systems level comparison among co-expression networks, the filtering and preprocessing of the data for zero variance led to elimination of 36 and 528 genes from high chill and low chill condition datasets, respectively, and 16001 shared genes were used for further analyses. Positive correlation and significant p-values of average gene expression (corr = 0.95, p ≤ 1e–200) (Supplementary Fig. S4) ensured the comparability of these datasets. From the hierarchical clustering based on DisTOM, a total of 28 modules were obtained for each dataset at an appropriate deepsplit of 2 to have comparable number of modules (Supplementary Fig. S5). The analysis of network module preservation statistics yielded Z_summary_ statistics by comparing low chilling (test dataset) modules against corresponding high chilling (control dataset) modules (Table 1 and Fig. 6). The modules with Z_summary_ statistics ≤3.0, were considered as significantly non-conserved.

Further the consensus network analysis revealed that all selected non-conserved modules based on Z_summary_ statistics (yellow, darkorange, darkgrey, orange, white, and purple) had have very poor overlapping of genes with modules from test dataset (Supplementary Fig. S6). The number of significant modules was further reduced to only two (yellow and purple) based on maximum number of significantly enriched terms. Additionally, heat-maps of both modules were clearly differentiating their expression behaviour between high chill and low chill datasets, particularly the ST, GT and FS stages have differential expression (Supplementary Fig. S7). In STH and FSH stages, the genes of yellow and purple modules were found to have down-regulated expression as compared to STL and FSL, respectively. While in contrast, these genes were found to be up-regulated in GTH as compared to GTL.

The GO enrichment analysis of yellow and purple modules showed common enrichment of post-embryonic development and some other cellular metabolic processes. The top five “biological processes” category of GO terms enriched for both modules are listed in Table 2. The important biological processes GO terms enriched in yellow module were post-embryonic development, cellular process, multicellular organismal process, protein modification process, reproductive process, reproduction, reproductive developmental process, reproductive structure development and fruit development. Similarly, purple module was observed to be enriched with terms like, post-embryonic development, cellular response to stimulus, cellular catabolic process and macromolecule localization. Network topology analysis of these two modules (yellow and purple) was performed using Cytoscape software to obtain hub genes using degree centrality measure (Supplementary Fig. S8). Genes at a threshold of intra-modular connectivity (*K*_*i*_), *K*_*i*_ ≥ 0.9 were selected and top 5 genes based on *K*_*i*_ were mapped to the network of each module. Interestingly, most of such genes with high connectivity were either found to specifically interact with hub genes or itself forming the hub genes (Table 3, Supplementary Fig. S8).

## Discussion

Winter dormancy in temperate perennial plants plays a crucial role in surviving adverse environmental conditions of winters. In apple, availability of sufficient chilling during dormancy period has been found to be positively correlated with fruit productivity^21^,^23^,^24^,^25^. Therefore, it is important to understand molecular mechanism of chilling mediated regulation of dormancy release and subsequent fruit set in apple. Recently Porto *et al*.^10^ used microarray to identify the transcriptional changes associated with chilling acquisition in dormant apple bud, however, the effect of chilling acquisition during dormancy on transcriptional changes associated with subsequent active growth and fruit set in apple under field conditions has not been studied, so far. Thus in the present study, transcriptional profiling during dormant and active growth stages under differential chilling was performed using RNA-seq approach. The influence of differential chilling has been observed through difference in DEGs (Fig. 2 and Supplementary Table S1). The results revealed that higher chilling is associated with the higher number of DEGs during active growth and subsequent fruit set stages.

The role of ABA in dormancy and its maintenance has been previously reported in plants. Dormancy release in leafy spurge^26^, and pear^5^ has been reported to be associated with decrease in the endogenous levels of ABA. The expression of transcripts related to ABA synthesis and its signaling were found to be high in dormant buds of poplar^27^. Similarly, hydrogen cyanamide mediated dormancy break in grape buds was found to be mediated through ABA metabolism and associated with high expression of *ABA 8*′*-hydroxylase* and increased ABA catabolites level^28^. Although, the ABA level was found to be reduced in both, chilled and non-chilled apple buds during dormancy release, the level of ABA was more in chilled buds as compared to non-chilled buds^23^,^29^. Similarly in the present investigation, the lower expression of the ABA biosynthetic gene *NCED3* and higher expression of ABA catabolic gene *ABA 8*′*-hydroxylase* suggested that low chill conditions might be associated with lower ABA contents during bud break and subsequent fruit set. Taken together, these results suggest that chilling might affect the bud break process by regulating the ABA metabolism, where high ABA level under high chill conditions might have further role to encounter the low temperature stress.

The gibberellic acid (GA) is also known to play important role during dormancy in plants. Recently, the exogenous application of GA was found to substitute the chilling requirement in *Populus*. Moreover, the transcript abundance of GA biosynthetic gene, *GA 20-oxd* was increased along with the chilling acquisition, while catabolic gene, *GA 2-oxd* was upregulated towards the end of chilling period^14^. In contrast, decreased expression of *GA 20-oxd* and increased expression of *GA 2-oxd* was reported in Japanese pear, towards the dormancy release^5^. In the present study, increase in *GA 20-oxd* and decrease in *GA 2-oxd* was observed towards the apple bud break. However, under low chill conditions, the comparative low expression of *GA 20-oxd* and higher expression of *GA 2-oxd* during dormancy release, indicate that low chilling might be associated with low GA biosynthesis. Also, the higher expression of *DELLA* transcripts, a negative regulator of GA signaling^30^, during bud break and fruit set under low chill conditions, suggest the negative feed-back regulation of GA biosynthetic genes. Interestingly, beside the low expression of GA biosynthetic genes under low chill conditions, the higher expression of *PRE1*, a GA inducible gene with possible role in GA response^31^, suggests more GA responsiveness. This might be due to the fact that low chill condition was associated with low ABA biosynthesis, which has antagonistic effect to the GA response in numerous physiological processes^32^.

The involvement of ethylene has been known in several physiological processes. Exogenous application of ethylene was found to induce the dormancy in *Chrysanthemum*, while ethylene insensitive transgenic was unable to undergo dormancy even after exogenous ethylene application^33^. The higher transcript abundance of ethylene related genes was observed during dormancy induction in leafy spurge^26^. Similarly in *Populus*, the transient increase in ethylene related genes was observed during dormancy induction in response to short day photoperiod^27^. In contrast, dormancy release in Japanese pear and HC (hydrogen cyanamide) treatment in grapes has been found to be associated with increased transcript abundance of ethylene biosynthesis genes^5^,^15^. In the present study, the expression of *ACS* was found to be increased during bud break. While *ACO*, a gene encoding key enzyme of ethylene biosynthesis, was found to be down-regulated towards bud break. These results suggest that ethylene biosynthesis decreased towards the bud break, where the low chilling was found to be associated with low abundance of ethylene biosynthetic genes. Moreover, it was suggested that ethylene plays a role in ABA accumulation through up-regulation of the ABA biosynthetic gene *NCED*^34^. Similarly in the present study, lower expression of ethylene biosynthetic genes as well as *NCED3* under low chill conditions, might suggest a cross talk between ethylene and ABA levels under low chill conditions.

In apple, auxin has been suggested to play inhibitory role in dormancy release of lateral buds^18^. Moreover, the auxin was found to negatively regulate the cytokinin biosynthesis by controlling the expression of *cytokinin synthase*, a key gene involved in cytokinin biosynthesis^35^. On the contrary, in the present study, the expression of *cytokinin synthase* was also found to be higher under low chill conditions beside the higher expression of genes involved in auxin biosynthesis and auxin signaling. These results indicate that the transport of auxin to the stem region might be responsible for high expression of *cytokinin synthase*. Moreover, the high expression of *cytokinin synthase* suggests the high accumulation of cytokinin which might result in early bud break under low chill conditions.

In Arabidopsis, prolonged cold treatment results in flower initiation, a process known as vernalization^2^. The expression of flower promoting genes *FT* and *SOC1* was negatively regulated by *FLC*, which in turn are epigenetically downregulated by extended cold treatment^36^. The phenomenon of vernalization somewhat resembles dormancy in temperate plants, where extended cold treatment also causes the release of dormancy. However, the role of *FLC* in woody perennial plants is still obscure due to absence of close orthologs of Arabidopsis *FLC* gene in woody plants^37^,^38^. In the present investigation, the *FLC-*like transcript was found to exhibit higher expression in DBH as compared to DBL, which was further downregulated with the dormancy release. Similarly, the upregulation of *FLC*-like gene during chilling acquisition in apple buds was also reported^10^. The analysis of *EVG* locus of evergrowing mutant of peach revealed that the deletion of four out of six tandemly repeated *SVP-*like *DAM* genes was responsible for non-dormant phenotype^6^. Similarly, the downregulated expression of *DAM* genes was found to be associated with dormancy release in various perennial plants^9^,^39^,^40^. In the present study, downregulation of *DAM* genes (*MDP0000259294, MDP0000322567* and *MDP0000527190*) with bud break indicates the dormancy-associated expression of apple *DAM* genes, similar to that of other perennial plants. The comparative expression of *DAM* genes showed variation under differential chilling conditions, with higher expression of *DAM* genes under high chill conditions as compared to low chill conditions, which concomitantly decrease with the bud break. Similarly, the initial higher expression of *DAM* genes in Japanese pear buds was observed during early chilling accumulation and thereafter concomitant decrease in the expression of *DAM* genes was observed with the chilling accumulation^41^. Therefore, availability of differential chilling during dormancy appears to regulate the expression of *DAM* genes. In leafy spurge, the presence of CBF binding sites in the promoter region of *DAM* genes was found to be responsible for its low temperature inducible expression^7^. Therefore, *DAM* genes appear to play important role in chilling mediated bud dormancy regulation in apple.

In Arabidopsis, the *FT* was found to move from leaf to shoot apex either as mRNA or protein, where it acts as flower inducing signal^42^. The *SVP* and *FLC* in Arabidopsis negatively regulate *FT* to repress the flowering initiation^43^. Previously, the higher expression of *FT*-like genes, *MdFT1* and *MdFT2* was reported in the apical bud and reproductive tissues of apple, respectively, as compared to leaf tissue^44^. Moreover, the transgenic apple overexpressing *MdFT1* showed early flowering phenotype. In the present investigation, expression of *FT*-like gene was found to be gradually increased towards bud break, where low chill condition was associated with higher expression of *FT*-like gene as compared to high chill condition. Moreover, expression of *FT*-like gene was found to be antagonistic to that of *DAM* genes, which suggests that *DAM* might negatively regulate the expression of *FT*-like gene in apple. In a recent study, the antagonistic expression of *DAM* and *FT*-like genes in apple has been reported by our group^45^. Similarly, antagonistic expression of *DAM* and *FT*-like genes was reported in leafy spurge and Japanese pear^7^,^26^,^41^,^46^. The antagonistic expression of *DAM* and *FT*-like genes indicate their role in dormancy release and flowering initiation regulation in apple. A recent study reported detection of quantitative trait loci (QTLs) associated with chilling mediated bud break and flowering time regulation in apple, which was found to be co-localized with *DAM* and *FT* genes^47^. Another member of PEBP family, the *TFL1* in Arabidopsis, is involved in the suppression of floral induction through negative regulation of floral meristem identity genes *LEAFY* and *APETALA1*^48^. The transgenic Arabidopsis overexpressing apple *MdTFL1* gene, exhibit delayed flowering phenotype similar to *TFL1* overexpressing Arabidopsis lines, which suggest analogous function of *MdTFL1* to Arabidopsis *TFL1*^49^. Moreover, the antisense suppression of *MdTFL1* in apple exhibit precocious flowering with short juvenile phase, which reduced plant vigour due to cessation of vegetative growth, therefore, it was suggested that *MdTFL1* affects the plant architecture as well as flower development in apple^50^. In Japanese pear, the expression of *PpTFL1-1α* was found to be higher in buds which received higher chilling duration^41^. Similarly in present study, the expression of *TFL1*-like gene was found to be downregulated under low chill conditions as compared to high chill conditions, which might result into precocious flowering that might affect the plant vigour under low chill conditions.

The role of epigenetic control in the downregulation of *FLC* after extended cold exposure to promote flowering has been well established in Arabidopsis^51^,^52^,^53^. Similarly, downregulation of peach *DAM6* with concomitant histone methylation and deacetylation in its promoter region, corroborate the analogous function of *DAM* and *FLC*^9^. In the present study, expression of *FLC*-like and *DAM* transcripts was also found to be strongly upregulated in dormant bud, thereafter downregulated during bud break. Therefore, these results indicate that downregulation of *FLC*-like and *DAM* genes in apple might also involve similar epigenetic mechanism. Moreover, the increased expression of *Histone acetyltransferase1 (HAC1*) transcripts towards the initiation of active growth under high chill condition (Fig. 5), might be associated with transcriptional activation through histone acetylation^54^. However, the delayed flowering in Arabidopsis *HAC1* mutant has been related to higher expression of *FLC*, suggesting the histone acetylation of a gene acting as a repressor of FLC expression^55^. In hybrid aspen, histone deacetylase (*HDA*), *HDA08* and *HDA14* were found to be upregulated during dormant period^56^. In the present study, the *HDA08* and *HDA06* were found to be downregulated towards the dormancy release and fruit set, while in contrast, *HDA14* and *HDA19* were found to be upregulated towards the dormancy releases and initiation of active growth. The expression of histone acetylases and deacetylases during dormancy release and fruit set suggested the dynamic balance in their opposing activities to attain appropriate transcriptional level of target genes. The counterbalance between these activities has also been suggested previously^57^.

In addition, the DNA methylation level has been reported to be higher in dormant state as compared to active growth phase in chestnut^58^ and strawberry^59^. In our recent study, we reported decrease in transcript level of *de novo* DNA methyltransferase, *DRM2* during apple bud break^60^, where the basic transcript level of *DRM2* was higher under low chill condition. Also the basic transcript level of *MET1* and *CMT3* was found to be higher under low chill conditions as compared to high chill conditions during bud break. The similar increase in expression of peach maintenance DNA methyltransferase, *PpMET1*, in lateral bud meristem, was suggested to be associated with DNA replication in actively proliferating meristematic cells to maintain the DNA methylation pattern in cell lineages^61^. Moreover, we also observed comparative upregulated expression of *DME*-like and *ROS1*-like transcripts under high chill conditions which was found to corroborate the reduction in DNA cytosine methylation during bud break under high chill condition^60^. The downregulation of DNA glycosylase, *DME*-like in hybrid aspen during dormancy induction^56^, also supports our observation. Therefore, these results indicate that epigenetic control through cytosine methylation and histone acetylation plays an important role in regulating chilling mediated dormancy release in apple.

The co-expression network analysis reveals the differential behavior of two comparable datasets under differential chilling conditions during winter dormancy in apple. From the total identified six non-conserved high chill associated modules, only yellow and purple modules showed significant GO terms enrichment and also clearly differentiated their expression behavior under differential chilling conditions (Supplementary Fig. S8). The criterion of selecting only significant modules, while leaving the non-significant modules has also been previously used by several studies to identify the biologically meaningful modules^62^,^63^,^64^. The GO term enrichment analysis of yellow and purple modules showed the enrichment of post-embryonic development in both modules. The post-embryonic development of the shoot occurs during three distinguished temporal phases: juvenile vegetative phase, adult vegetative phase and reproductive phase^65^. In perennial plants including apple, post-embryonic development occurs during the initiation of flowering and flower bearing shoots from lateral dormant meristem after dormancy^66^. In the present analysis, chilling availability during dormancy period affects genes involved in post-embryonic development of the dormant buds in active growth phase. Therefore, it is apparent that molecular mechanism involved in vigorous re-initiation of meristematic activity after the winter dormancy might be modulated by chilling availability during winter dormancy.

Moreover, the hub genes identified in yellow and purple modules might frequently interact with other genes involved in specific pathway. The hub gene from the yellow module, *EARLY FLOWERING 7 (ELF7*) has been shown to be involved in flowering time regulation by regulating the expression of *FLC* in Arabidopsis^53^. Similarly, the gene with high inter-modular connectivity in yellow module, *RAF10*, a mitogen-activated protein kinase kinase kinase (MAP3K), has been reported to act as positive regulator of ABA response and seed dormancy in Arabidopsis^67^. In case of purple module, one of the hub genes encoding F-box family protein has been known to be involved in degradation of cell cycle regulatory proteins^68^ and in floral development^69^,^70^. The gene with high inter-modular connectivity in purple module, *ZEP4*, encodes a zinc finger protein was suggested to negatively regulate ABA signalling during seed germination in Arabidopsis^71^. Recently, the modification of ABA metabolism has also been suggested to play important role during dormancy regulation in grapes^28^ and peach^72^. Therefore, the role of hub genes, identified through network analysis, in regulating cell cycle, ABA metabolism and flowering time etc., might suggest their importance in regulating winter dormancy in apple.

## Conclusions

In the present study, transcriptome analysis of samples collected during bud break and fruit set in apple under differential chilling availability in field conditions has been carried out. The higher number of DEGs during active growth phases under high chill condition, suggests that chilling availability drives the transcriptional changes during dormancy release. It is apparent that, chilling availability affects the expression of genes involved in regulation of phytohormones and epigenetic mechanism, such as DNA methylation and histone modification. The expression profiles of *DAM, FLC*-like, *FT*-like and *TF1*-like genes suggest that early flowering was induced under low chill condition. The plant has to prepare itself (for example resource generation and allocation) before entering into the reproductive phase, therefore early flowering might adversely affect the plant vigour in the reproductive phase of the phenological cycle. In addition, the co-expression network analysis revealed the presence of chilling associated gene modules. The enrichment of “post-embryonic development” for two high chill specific modules, indicates their involvement in vigorous re-initiation of meristematic activity in bud tissue during dormancy release, under high chill condition.

## Materials and Methods

### Plant materials

Plant material was collected from apple trees of Royal delicious cultivar, selected from two apple orchards situated at Palchan (32° 18′36″N, 77° 10′40 E″; altitude 2350 masl) and Seobag (31° 58′57″N, 77° 07′43E″; altitude 1250 masl) of Kullu district, Himachal Pradesh (HP), India. From each selected location, the samples of four developmental stages, namely dormant bud (DB), silver tip (ST), green tip (GT) and initial fruit set (FS) were collected during January 8, 2013 to April 15, 2013 from Seobag and during January 8, 2013 to May 8, 2013 from Palchan (Fig. 1). Each sample was the tissues collected from three different trees and immediately frozen in liquid nitrogen and stored at −80 °C for further use. The temperature data depicting difference in chilling availability for both locations were obtained from nearby data recording centers; Hill Agricultural Research & Extension Centre, Kullu (HP) for Seobag (low chill) and DRDO-Snow and Avalanche Study Estt. station Bahang, Manali (HP) for Palchan (high chill), for November to April months of three consecutive years 2011–12, 2012–13 and 2013–14. The accumulated chilling units for each collected samples were calculated using Utah model^73^.

### RNA sequencing and data analysis

Total RNA from each plant sample was extracted as described previously^60^. The total RNA was quantified using Nanodrop specrtophotometer and RNA integrity was checked on denaturating formaldehye agarose gel and Agilent 2100 Bioanalyzer. The purified mRNA from each sample was used to prepare one RNA-seq library using TruSeq RNA sample preparation kit v2 (Illumina Inc., USA) following manufacturer’s instructions. The PCR enriched libraries were quantified by fluorescence based Qubit assay, and further insert size validation was performed using Agilent 2100 Bioanalyzer. The clonal clusters for 10 pM of each library (representing one sample) were generated in the flow cell using TruSeq PE Cluster Kit v5 on cluster station (Illumina Inc., USA) and paired end sequencing (2 × 76) on Genome analyzer IIx was performed (Illumina Inc., USA). The raw reads generated from Illumina GAIIx were submitted as BioProject (PRJNA306594) to NCBI Sequence Read Archive under accession number SRP067619. The raw reads were filtered for removing low quality sequences using NGS QC toolkit^74^. All the remaining high quality reads were *de novo* assembled using CLC Genomics Workbench 6.5 (http://www.clcbio.com). Various parameters like K-mer and N50 were optimized to get the best assembly results. The assembled contigs were annotated against non-redundant protein database of NCBI using BLASTX program. The assembled contigs were also subjected to GO and KEGG analysis using AgriGO^75^ and KAAS^76^, respectively. The expression of contigs was calculated in terms of FPKM (fragments per kilobase of exon per million fragments mapped). The differentially expressed genes (DEGs) with p-value < 0.05 and log2 fold change >2 were identified using EdgeR^77^. The identified DEGs were further subjected to GO and KEGG enrichment analysis using AgriGO and KOBAS^78^, respectively. The enriched GO terms and KEGG pathways with p-value ≤ 0.05 were considered as significantly enriched for DEG sets.

### cDNA preparation and qRT-PCR analysis

The 1 μg of total RNA was converted to first strand cDNA using RevertAid H minus cDNA synthesis kit (Fermentas), following the manufacturer’s instructions. The primers for qRT-PCR analysis were designed using Primer Express software version 3.0.1 (Invitrogen) with default parameters. The qRT-PCR assays were performed with 1:10 diluted cDNA in 20 μl reaction using SYBR green PCR master mix (Thermo Scientific). The relative fold change in the expression of target genes was calculated by comparative delta-delta Ct method using GAPDH as internal control genes for qRT-PCR data normalization^79^. The primer sequences of all the genes used for validation of expression levels as obtained by RNA-seq data are given in Supplementary Table S4.

### Network construction and enrichment analysis

Normalized RNA-seq datasets of apple under four developmental conditions were analyzed through network biology approach to explore their differential behavior towards high- and low-chill conditions. Top hit BLASTX annotation for the complete transcriptomic sequences against Arabidopsis proteome, at the e-value cutoff of 1e–05, was considered for network analysis. Transcripts with identical TAIR identifier were analyzed to obtained unique transcripts with maximum statistical variance^80^. Transcripts were corrected for excessive missing values and sample outliers, and were removed while performing pre-processing of both datasets using ‘Weighted Gene Co-Expression Network Analysis’ (WGCNA) library of R statistical package v3.0.1^81^. For comparative analysis of expression data, common transcripts were considered to construct separate gene co-expression network for each dataset that were further probed to elucidate differential behavior of transcripts during dormancy release and fruit set under low and high chill conditions. For each weighted network, Pearson correlation matrices corresponding to gene expression were computed, which were further transformed into matrices of connection strengths using a power function that best suits its scale-free behavior. These connection strengths were transformed into a topological overlap similarity measure (TOM) using equation (1), which was further used to compute dissimilarity TOM (DistTOM) as in equation (2)^82^. DistTOM similarity measure between two genes (*i* and *j*) is described as:


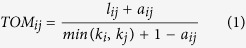



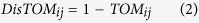


where, 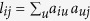, and 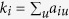 is the node connectivity and *a*_*ij*_ is the network adjacency. Finally, hierarchical clustering along with DistTOM measure was used to obtain comparable modules from both datasets with appropriate value of deepsplit^81^. Module preservation (on module-by-module basis) analysis, was implemented to calculate Z_summary_ score using module preservation function in the WGCNA library^83^. Module definitions from the reference network (high chill) were mapped on the test network (low chill). Module preservation was also quantified in terms of significant overlap in genes, using overlap table function^84^, by comparing test and reference networks. Modules with less conservation profile were obtained, which may be responsible for the differential behavior during dormancy releases under differential chilling conditions.

Functional annotations of the non-conserved modules were performed using AgriGO and KAAS. Cytoscape v3.0 was used to visualize the co-expression networks^85^, and was analyzed for topological parameters using Network Analyzer, a Java plugin for Cytoscape. Key genes, responsible for differential behavior in both datasets, were identified based on high degree (hub genes: gene with higher number of connection) as well as genes having high intramodular connectivity for selected modules. Hub genes are thought to be key genes of a set of co-expressed genes; therefore, identification of such hub genes associated GO terms and pathways provide insight into the differential mechanisms carried out under different chilling conditions.

## Additional Information

**How to cite this article**: Kumar, G. *et al*. Chilling Affects Phytohormone and Post-Embryonic Development Pathways during Bud Break and Fruit Set in Apple (*Malus domestica* Borkh.). *Sci. Rep.* **7**, 42593; doi: 10.1038/srep42593 (2017).

**Publisher's note:** Springer Nature remains neutral with regard to jurisdictional claims in published maps and institutional affiliations.

## Supplementary Material

Supplementary Information

## Figures and Tables

**Table 1:** Z_summary_ statistics for the conservation of the modules.

Sl. No.	Modules	Module Size	Z _summary_
1	greenyellow	400	15.6346343
2	blue	400	12.35839264
3	magenta	400	11.79187395
4	tan	400	11.64093742
5	brown	400	11.33590755
6	cyan	400	10.6234039
7	salmon	400	10.56140266
8	red	400	9.99823054
9	midnightblue	400	9.85889887
10	pink	400	9.61736237
11	lightcyan	395	8.51593021
12	lightgreen	220	8.28904928
13	turquoise	400	7.95152399
14	royalblue	168	7.80708899
15	grey	125	6.70819826
16	darkgreen	155	5.51287319
17	grey60	368	5.40965638
18	green	400	5.3351691
19	lightyellow	199	5.18543768
20	darkturquoise	115	5.01684533
21	black	400	4.69337278
22	darkred	167	4.48795744
23	yellow	400	2.50318424
24	darkorange	59	1.90620282
25	darkgrey	94	1.27831327
26	orange	84	0.08246996
27	white	49	−0.53279785
28	purple	400	−1.05107155

**Table 2:** Table depicting the GO terms enriched in yellow and purple modules.

GO term	Description	p-value	FDR
Yellow module			
GO:0009791	post-embryonic development	1e-14	2.4e-11
GO:0009987	cellular process	7.3e-13	8.4e-10
GO:0032501	multicellular organismal process	1.8e-07	0.00014
GO:0006464	protein modification process	3.2e-07	0.00018
GO:0044237	cellular metabolic process	4e-07	0.00018
**Purple module**			
GO:0009791	post-embryonic development	7.1e-05	0.081
GO:0051716	cellular response to stimulus	0.00021	0.082
GO:0044248	cellular catabolic process	0.00017	0.082
GO:0033036	macromolecule localization	0.00035	0.093
GO:0033554	cellular response to stress	0.00048	0.093

**Table 3:** List of hub genes and genes with high intra-modular connectivity in yellow and purple modules.

Yellow module		
**Hub genes**		
Contig_17248	AT2G28800.4	ALB3/ALBINO 3
Contig_18725	AT1G79730.1	EARLY FLOWERING 7, ELF7
Contig_44629	AT4G15417.1	ATRTL1, RNASE II-LIKE 1, RTL1
Contig_9114	AT4G01810.1	Sec23/Sec24 protein transport family protein
Contig_21105	AT3G57050.1	CBL, CYSTATHIONINE BETA-LYASE
**Genes with high intra-modular connectivity**		
Contig_23277	AT3G58090.1	Disease resistance-responsive family protein
Contig_30715	AT3G07860.1	Ubiquitin-like superfamily protein
Contig_39306	AT5G49470.1	RAF10
Contig_9824	AT1G11310.2	MILDEW RESISTANCE LOCUS O 2, MLO2, PMR2
Contig_12255	AT5G66100.1	ATLARP1B, LA RELATED PROTEIN 1B
**Purple module**		
**Hub genes**		
Contig_14561	AT3G18640.1	Zinc finger protein (Histone methyltransferase)
Contig_11757	AT3G26890.6	unknown protein
Contig_18298	AT2G39780.1	RIBONUCLEASE 2, RNS2
Contig_48245	AT1G30920.1	F-box family protein
Contig_28979	AT1G17680.2	TPR-containing protein
**Genes with high intra-modular connectivity**		
Contig_48245	AT1G30920.1	F-box family protein
Contig_13529	AT1G23260.1	UBIQUITIN E2 VARIANT 1 A
Contig_11757	AT3G26890.6	unknown protein
Contig_11772	AT5G35980.2	YAK1, YEAST YAK1-RELATED GENE 1
Contig_27146	AT1G66140.1	ZFP4, ZINC FINGER PROTEIN 4
