# Supplementary material for: Chilling Affects Phytohormone and Post-Embryonic Development Pathways during Bud Break and Fruit Set in Apple (Malus domestica Borkh.)
Source: Sci Rep. 2017 Feb 15;7:42593. doi: 10.1038/srep42593 (PMC5309832; doi:10.1038/srep42593)
Supplement: Supplementary Information [file srep42593-s1.pdf]

Supplementary Information:

Chilling Affects Phytohormone and Post-Embryonic Development Pathways during Bud Break and Fruit Set in Apple (*Malus domestica* Borkh.)

Gulshan Kumar[1,2], Khushboo Gupta[1,†], Shivalika Pathania[1], Mohit Kumar Swarnkar[1], Usha Kumari Rattan[1], Gagandeep Singh[1], Ram Kumar Sharma[1], Anil Kumar Singh[1,2,†]

[1]Department of Biotechnology, CSIR-Institute of Himalayan Bioresource Technology, Palampur, 176061, India

[2]Academy of Scientific and Innovative Research, New Delhi

[†]Present Address: ICAR-Indian Institute of Agricultural Biotechnology, PDU Campus, IINRG, Namkum, Ranchi-834010 (JH), India

*Correspondence and request for material should be addressed to A.K.S. (email: anils13@gmail.com)

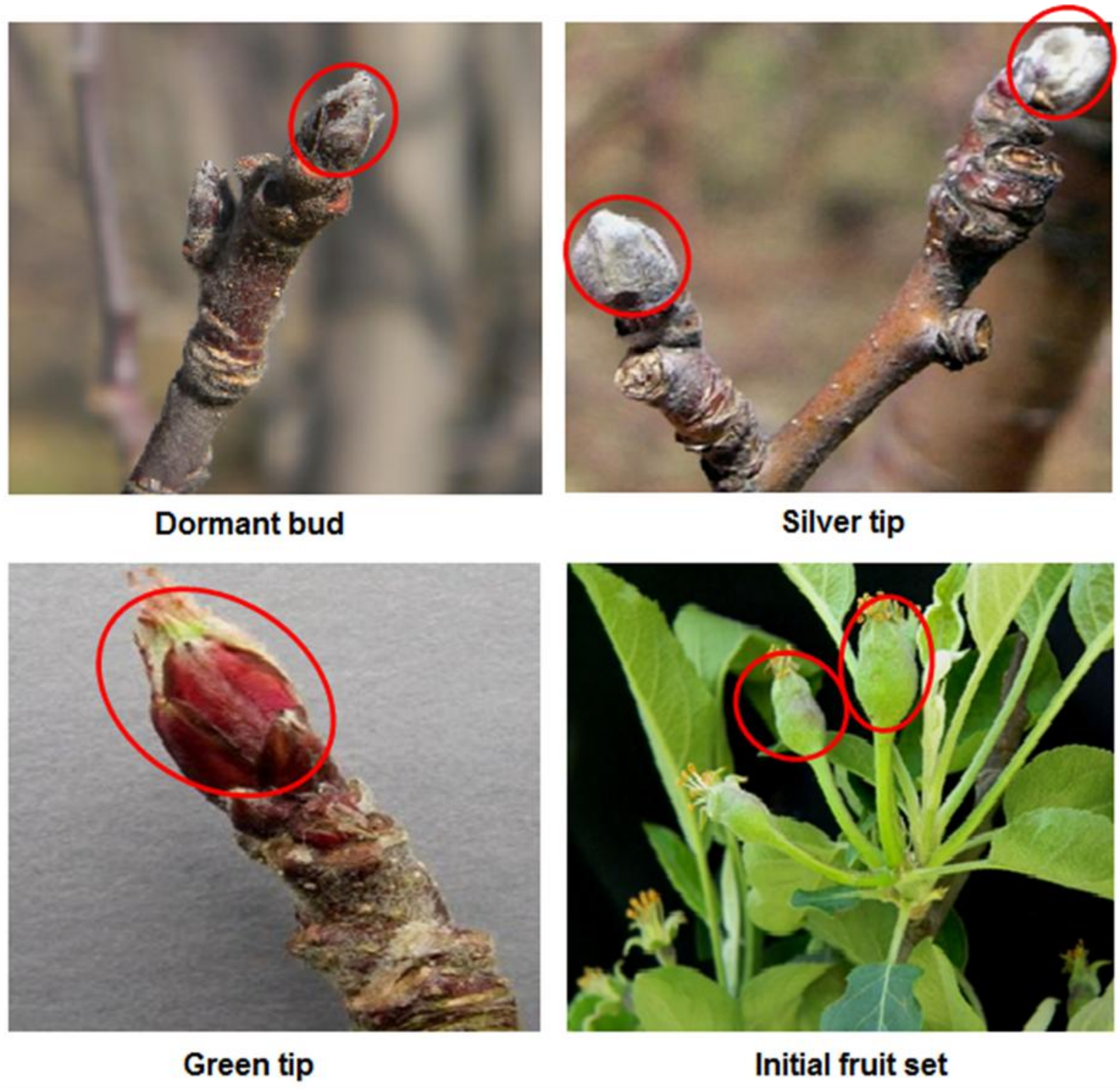

Supplementary Fig. S1 The pictorial representation of samples collected from the experimental site. The Dormant bud, Silver tip, Green tip and Initial fruit set represent the morphological characters of developmental stages collected for present analysis. The actual tissue excised as experimental sample is encircled with red color.

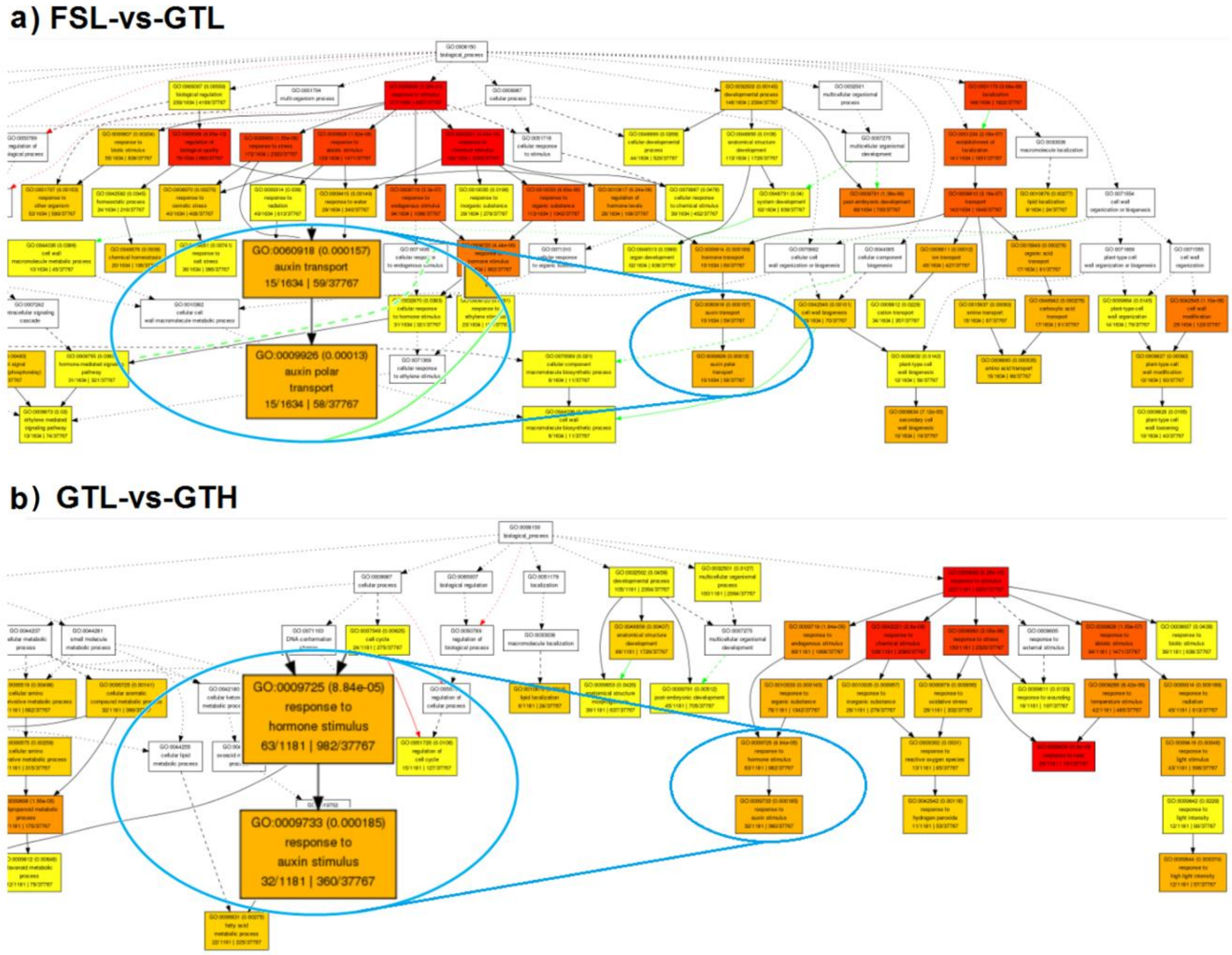

Supplementary Fig. S2 Hierarchical tree graph of enriched GO terms in biological process category highlighting auxin polar transport and auxin stimulus. The GO enrichment of FSL-vs-GTL (A) and GTL-vs-GTH (B) comparative conditions are shown.

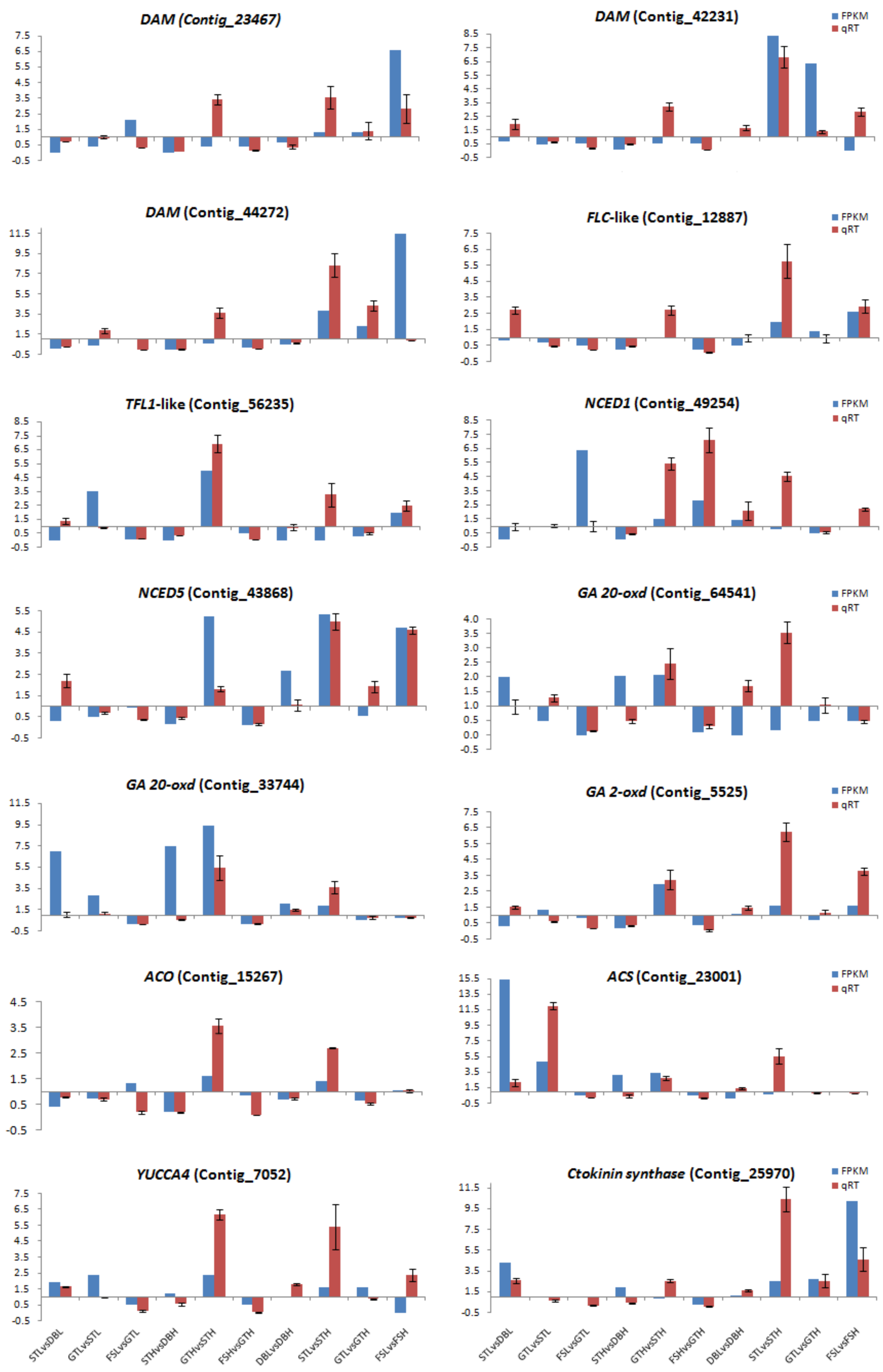

Supplementary Fig. S3 The validation of FPKM based relative expression of various floral time regulation and phytohormone metabolism related genes, by quantitative reverse transcriptase-polymerase chain reaction (qRT-PCR).

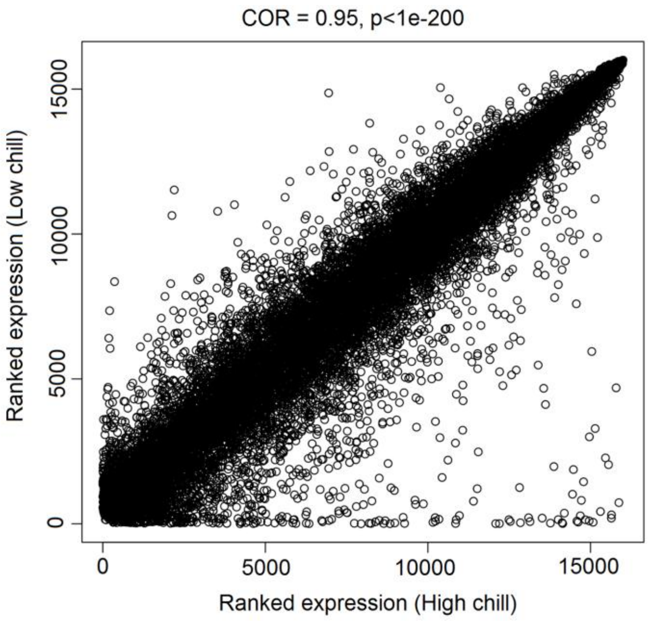

Supplementary Fig. S4 Ranked expression correlation between two datasets. Each dot represents a gene common between datasets, with X and Y axis represents gene's ranked expression in samples from high chill and low chill data sets, respectively.

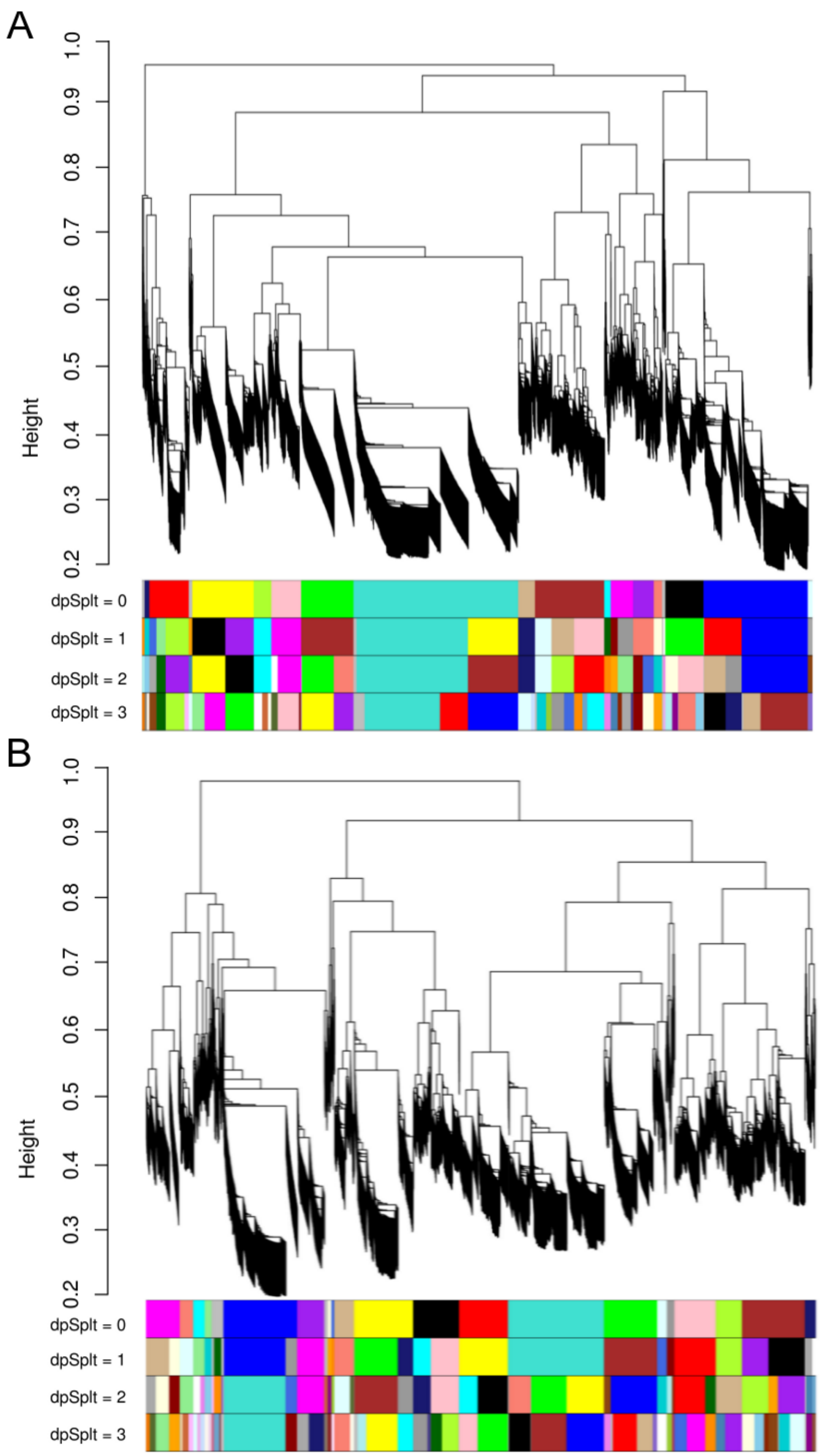

Supplementay Fig. S5 The deepsplit plot for identification of comparable modules in two datasets. The low chill dataset (A) and high chill dataset (B) show the presence of 28 gene modules.

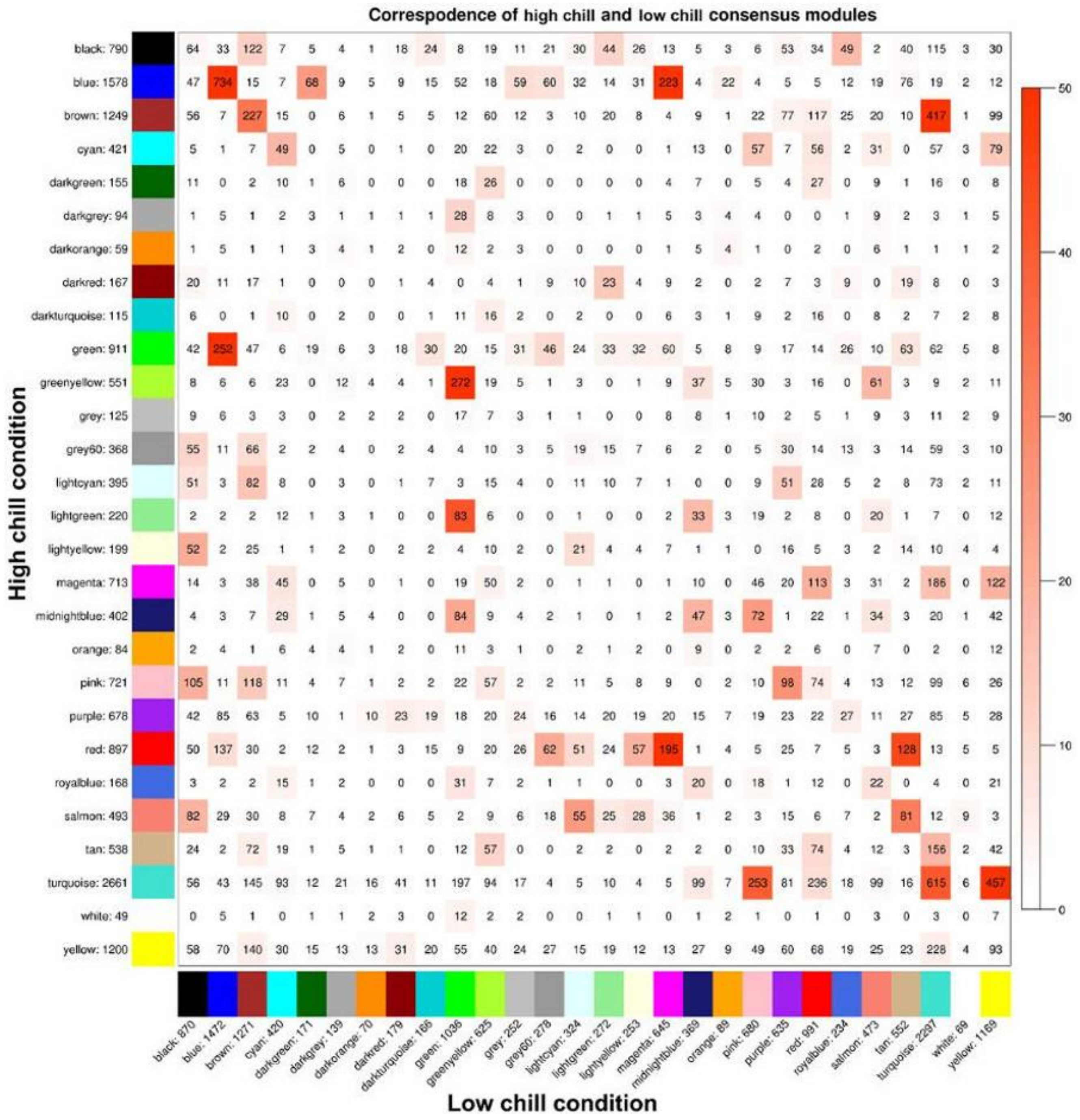

Supplementary Fig. S6 The consensus match for the modules under low and high chill conditions for overlapping genes.

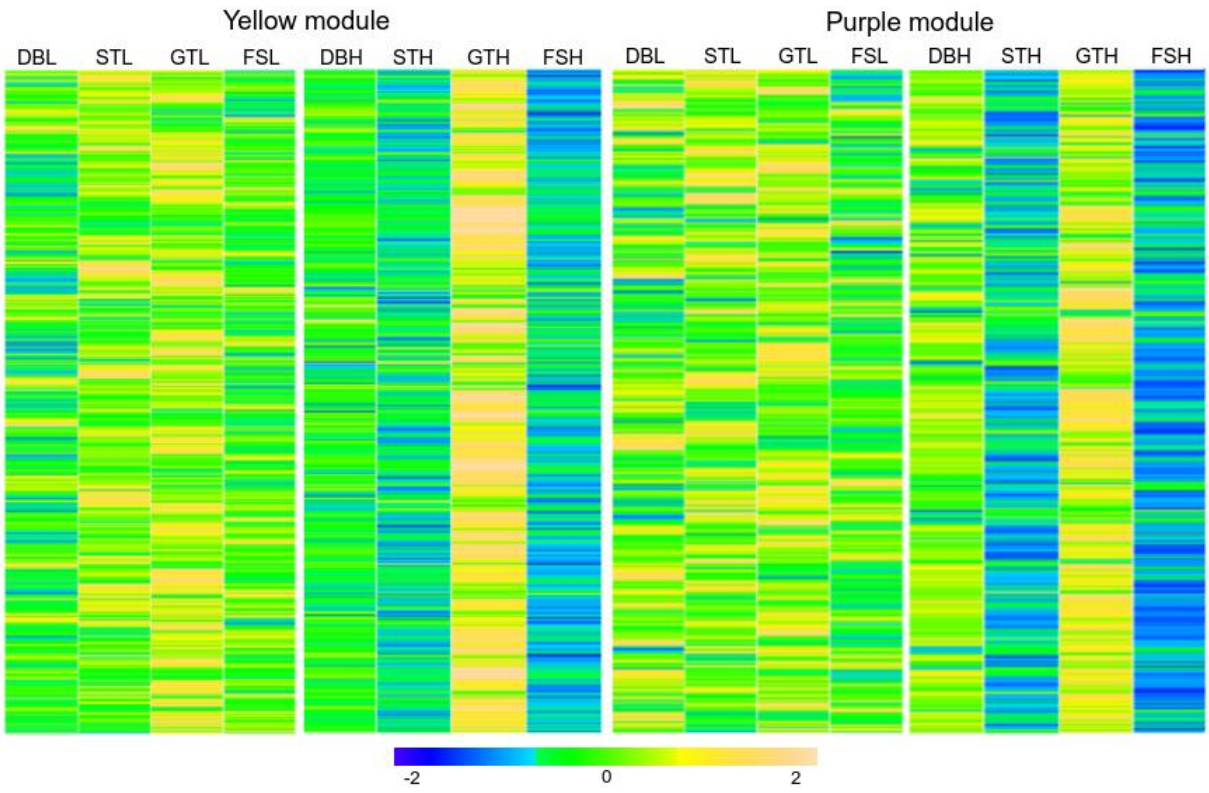

Supplementary Fig. S7 Heat map showing differential expression of genes during dormancy release under differential chilling conditions in yellow and purple module.

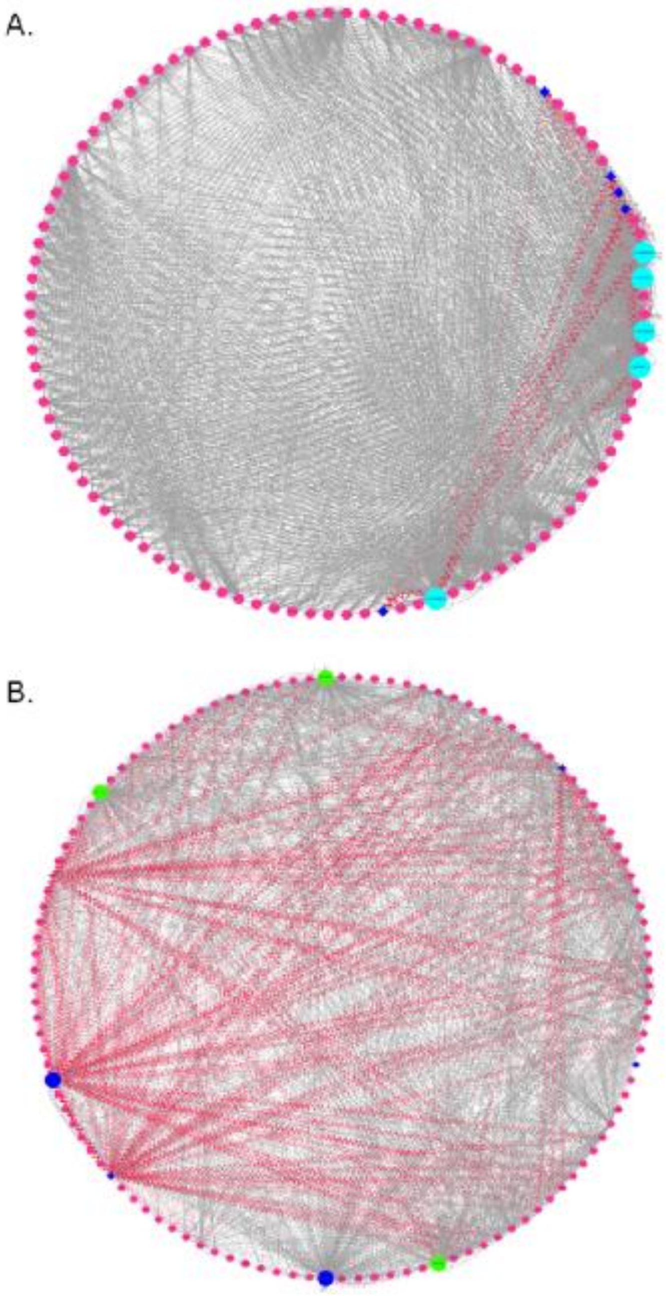

Supplementary Fig. S8 Network of selected modules. In yellow module network (A), the hub genes are shown in cyan color while the genes with high inter-modular connectivity are shown in blue color. In purple module network (B), the hub genes are shown in green and blue (large spheres) color, while the genes with high inter-modular connectivity are shown in blue color (small as well as large spheres).

Supplementary Table S1 Summary of differentially expressed genes (DEGs) under differential chilling conditions in various comparative conditions.

**Conditions**	**Comparative conditions**	**Total DEGs**	**Upregulated DEGs**	**Downregulated DEGs**
High chill	STH-vs-DBH	8457	3302	5155
	GTH-vs-STH	11825	7886	3939
	FSH-vs-GTH	12373	8212	4161
Low chill	STL-vs-DBL	8904	3477	5427
	GTL-vs-STL	5233	2506	2727
	FSL-vs-GTL	6219	3084	3135
Low chill versus high chill	DBL-vs-DBH	1325	697	628
	STL-vs-STH	2463	1437	1026
	GTL-vs-GTH	9487	2238	7249
	FSL-vs-FSH	2131	1147	984

Supplementary Table S2 The biological processes category of enriched GO term for the DEGs under low chill and high chill comparative conditions.

Comparative conditions	GO term associated with up-regulated DEGs		GO term associated with down-regulated DEGs	
**STL-vs-DBL**	GO:0050896	response to stimulus	GO:0009791	post-embryonic development
	GO:0009628	response to abiotic stimulus	GO:0048856	anatomical structure development
	GO:0006950	response to stress	GO:0032501	multicellular organismal process
	GO:0008152	metabolic process	GO:0006468	protein amino acid phosphorylation
	GO:0009791	post-embryonic development	GO:0032502	developmental process
**GTL-vs-STL**	GO:0044085	cellular component biogenesis	GO:0009791	post-embryonic development
	GO:0008152	metabolic process	GO:0009987	cellular process
	GO:0050896	response to stimulus	GO:0008152	metabolic process
	GO:0015979	photosynthesis	GO:0009628	response to abiotic stimulus
	GO:0009058	biosynthetic process	GO:0050896	response to stimulus
**FSL-vs-GTL**	GO:0009791	post-embryonic development	GO:0050896	response to stimulus
	GO:0032501	multicellular organismal process	GO:0042221	response to chemical stimulus
	GO:0007275	multicellular organismal development	GO:0065008	regulation of biological quality
	GO:0048856	anatomical structure development	GO:0008152	metabolic process
	GO:0009987	cellular process	GO:0009628	response to abiotic stimulus
**STH-vs-DBH**	GO:0050896	response to stimulus	GO:0009791	post-embryonic development
	GO:0009628	response to abiotic stimulus	GO:0009987	cellular process
	GO:0006950	response to stress	GO:0007049	cell cycle
	GO:0042221	response to chemical stimulus	GO:0008152	metabolic process
	GO:0009791	post-embryonic development	GO:0048856	anatomical structure development
**GTH-vs-STH**	GO:0008152	metabolic process	GO:0009791	post-embryonic development
	GO:0050896	response to stimulus	GO:0008152	metabolic process
	GO:0044237	cellular metabolic process	GO:0009987	cellular process
	GO:0009987	cellular process	GO:0032501	multicellular organismal process
	GO:0006950	response to stress	GO:0050896	response to stimulus
**FSH-vs-GTH**	GO:0008152	metabolic process	GO:0050896	response to stimulus
	GO:0009987	cellular process	GO:0006950	response to stress
	GO:0009791	post-embryonic development	GO:0009628	response to abiotic stimulus
	GO:0050896	response to stimulus	GO:0006629	lipid metabolic process
	GO:0044237	cellular metabolic process	GO:0008152	metabolic process
**DBL-vs-DBH**	GO:0055114	oxidation reduction	GO:0010876	lipid localization
	GO:0005976	polysaccharide metabolic process	GO:0006950	response to stress
	GO:0050896	response to stimulus	GO:0005975	carbohydrate metabolic process
	GO:0042546	cell wall biogenesis	GO:0050896	response to stimulus
	GO:0008152	metabolic process	GO:0019438	aromatic compound biosynthetic process
**STL-vs-STH**	GO:0050896	response to stimulus	GO:0009791	post-embryonic development
	GO:0006950	response to stress	GO:0010876	lipid localization
	GO:0009628	response to abiotic stimulus	GO:0006091	generation of precursor metabolites
	GO:0009987	cellular process	GO:0010166	wax metabolic process
	GO:0009791	post-embryonic development	GO:0009607	response to biotic stimulus
**GTL-vs-GTH**	GO:0050896	response to stimulus	GO:0008152	metabolic process
	GO:0009408	response to heat	GO:0050896	response to stimulus
	GO:0006260	DNA replication	GO:0006950	response to stress
	GO:0042221	response to chemical stimulus	GO:0044237	cellular metabolic process
	GO:0006950	response to stress	GO:0009987	cellular process
**FSL-vs-FSH**	GO:0009791	post-embryonic development	GO:0006091	generation of precursor metabolites
	GO:0032501	multicellular organismal process	GO:0008152	metabolic process
	GO:0007275	multicellular organismal development	GO:0015979	photosynthesis
	GO:0032502	developmental process	GO:0006412	translation
	GO:0048856	anatomical structure development	GO:0044237	cellular metabolic process

Supplementary Table S3 The top 5 KEGG pathways enriched for DEGs under low chill and high chill comparative conditions.

Comparative conditions	Pathways enriched among up-regulated DEGs	Pathways enriched among down-regulated DEGs
**STL-vs-DBL**	Plant hormone signal transduction	Fatty acid metabolism
	Ribosome	Arginine and proline metabolism
	Circadian rhythm - plant	Steroid biosynthesis
	Amino sugar and nucleotide sugar metabolism	mTOR signaling pathway
	Pentose and glucuronate interconversions	Glycolysis / Gluconeogenesis
**GTL-vs-STL**	Ribosome	Plant hormone signal transduction
	Carbon fixation in photosynthetic organisms	Pentose and glucuronate interconversions
	Fructose and mannose metabolism	Starch and sucrose metabolism
	Porphyrin and chlorophyll metabolism	Glycolysis / Gluconeogenesis
	Amino sugar and nucleotide sugar metabolism	Amino sugar and nucleotide sugar metabolism
**FSL-vs-GTL**	Ribosome biogenesis in eukaryotes	Ribosome
	Starch and sucrose metabolism	Carbon fixation in photosynthetic organisms
	Plant hormone signal transduction	Amino sugar and nucleotide sugar metabolism
	Cysteine and methionine metabolism	Galactose metabolism
	Fatty acid metabolism	Nitrogen metabolism
**STH-vs-DBH**	AMPK signaling pathway	Cell cycle
	Regulation of actin cytoskeleton	Ribosome
	Carbon fixation in photosynthetic organisms	Arginine and proline metabolism
	Cutin, suberine and wax biosynthesis	Carbon fixation in photosynthetic organisms
	Glycerolipid metabolism	Other glycan degradation
**GTH-vs-STH**	VEGF signaling pathway	Carbon metabolism
	Riboflavin metabolism	Starch and sucrose metabolism
	Diterpenoid biosynthesis	Glycine, serine and threonine metabolism
	Wnt signaling pathway	Fructose and mannose metabolism
	Sphingolipid signaling pathway	Phenylpropanoid biosynthesis
**FSH-vs-GTH**	ErbB signaling pathway	Ribosome
	Ras signaling pathway	Carbon fixation in photosynthetic organisms
	mTOR signaling pathway	Fructose and mannose metabolism
	Sphingolipid metabolism	Glycolysis / Gluconeogenesis
	Tryptophan metabolism	Glycerolipid metabolism
**DBL-vs-DBH**	Zeatin biosynthesis	Glycolysis / Gluconeogenesis
	Ribosome	Cutin, suberine and wax biosynthesis
	Starch and sucrose metabolism	Glycerolipid metabolism
	Other glycan degradation	Starch and sucrose metabolism
	Arginine and proline metabolism	Glycerophospholipid metabolism
**STL-vs-STH**	Plant hormone signal transduction	Phenylpropanoid biosynthesis
	PI3K-Akt signaling pathway	Starch and sucrose metabolism
	Starch and sucrose metabolism	Pentose and glucuronate interconversions
	MAPK signaling pathway	GABAergic synapse
	AMPK signaling pathway	beta-Alanine metabolism
**GTL-vs-GTH**	Plant hormone signal transduction	Ether lipid metabolism
	DNA replication	Valine, leucine and isoleucine biosynthesis
	AMPK signaling pathway	Ubiquinone and terpenoid-quinone biosynthesis
	Cell cycle	Oocyte meiosis
	Glutathione metabolism	beta-Alanine metabolism
**FSL-vs-FSH**	Phenylpropanoid biosynthesis	Ribosome
	Starch and sucrose metabolism	Oxidative phosphorylation
	Phenylalanine metabolism	Photosynthesis
	Plant-pathogen interaction	Plant hormone signal transduction
	Ribosome	Cyanoamino acid metabolism

Supplementary Table S4 Gene name, Contig sequence, primer sequence and amplicon size used for RNA-seq data validation using qRT-PCR. The sequence of amplicon has been highlighted with yellow colour.

Gene name	Contig sequence	Primer name and sequence	Amplicon size
DAM<br>(Contig_23467)	ATGAAGGGTGTGGACCTTGAAGACTTGGATCTGGATGAACTACAGAAGTTAGAAAAATTGGTGGAAGCAAGCCTTGG<br>CCGTGTGATTCAAACTAAGGAAGAAAAGATTATGAGTGAGGTTATGGCACTTGAGAAAAAGGGAGCTGAGCTGATAG<br>AAGCTAACAACCAGCTAAGCCACAGGATGGTGATGTATCCCAGAGGAGATATTGGACCAGAGGCCATCCTGGAGTTGG<br>AAAACCTGAATAATATTGGAGAAGAAAGCGTGACATCTGAATCAACCACAAATGTCACCACCTGCTCCAACAGCTCTC<br>TTTCCCTTGAAGATGATTGCTCCGACATCTTGTCTCTCAAACTGGGGCTTCCG	Dam1(F) 5'-TTGGAGAAGAAAGCGTGACATC-3'<br>Dam1(R) 5'-TGTCGGAGCAATCATCTTCAAG-3'	90
DAM<br>(Contig_42231)	ATGGCGAGGGAGAAGATTCAGATCAAGAAGATCGACAACGCGACAGCGAGGCAGGTGACCTTTTCCAAGAGAAGAA<br>ATGGGCTTCTGAAGAAAGCTTATGAGCTCTCCGTTCTCTGTGATGCCGATATTGCTCTTATCATCTTTTCTTCCACCGGA<br>AAGCTCTT	Dam2(F) 5'-AGGCAGGTGACCTTTTCCAA-3'<br>Dam2(R) 5'-TCCGGTGGAAGAAAAGATGATAA-3'	108
DAM<br>(Contig_44272)	TACCAAGGATGTGATTGCAAGGTACAAATCACATACTGGTGGGGAAAAATCGGATCAAATCACGCTTCACCAACTGCA<br>GTCGGAGAAAGAAAACACGATCAGGCTGAGTAAGGAACTTGAGGATAAGACCCGCAAGCTGAGGCATATGAAGGGT<br>GAGGACCTTCAAGACTTGGATCTGGATCAACTGAACAAGTTAGAAAAATTGGTGGAAGTAAGCATTGGCCGTGTAATA<br>AAAACTAAGGAAAAAAAGATAATGAGTGAGATTATGGCACTTACGAACAAGGGAGCTGAGCTTATAGAAGCTAACAA<br>CCAACTAAAGCAGAGGTTGGTGATGTTATCCGCTGGAGGAGATATCGAACCGGCGGCGATCATGGAGTTGGAAAACCT<br>GAATAATGTTGGAGAAGAA	Dam3(F) 5'-ACCAAGGATGTGATTGCAAGGT-3'<br>Dam3(R) 5'-ACTCAGCCTGATCGTGTTTTCTT-3'	108
***FLC*-like**<br>(Contig_12887)	CGGAGAAGCGGGCTGATCAAGAAGGCACGTGAGCTGTCGGTGCTCTGCGGGGTGGAGGTCGGCCTTGTCATCTTCTC<br>CACAAAGGGGAGGCTCTACGAGTTCTCACCCAGCTTGAAGAGGAATTAGATGCACTTTTAAGACAAACAAGATCGAG<br>AAAGACACAGCTGATGATGGAAAACCTTACAGCTCTTATCGAGACGGAAAAACAGCTGAARGAAGAGAAGCGTCTCA<br>TAGAAAATGAGGTAGAAGTTGTGATAGAAAAGCAAGTGAAAAACAAGTACTCACAGTCACTTGATGGCTTTAATTTAT<br>CAGATTGCAGCACTGAAKCTGAAGGAGCAAGCAGASCAAGGACTGAGCTGCCGACGAGGAACCGGATCAGCAGAGC<br>ACTTCCTCCGACTATAACA	FLC(F) 5'-AGAAGCGGGCTGATCAAGAA-3'<br>FLC(R) 5'-AACTCGTAGAGCCTCCCCTT-3'	98
***TFL1*-like**<br>(Contig_58315)	ATGTCGAGGACGACGGAGCCATTGACGGTAGGAAGAGTGATAGGAGAGGTTGTTGACATCTTCACCCCAAGTGTGAA<br>GCTGAATGTAGTTTACAATCCAAACAAGCAAGTTGCTAATGGACATGAGCTCATGCCTTCTGTCATTGCTGAAAAACCT<br>CGCGTCGACATCGGAGGTGAAGATATGAGGG	TFL1(F) 5'-CCCCAAGTGTGAAGCTGAATG-3'<br>TFL1(R) 5'-CGATGTCGACGCGAGGTT-3'	105
NCED1<br>(Contig_49254)	ATGACACCGCCGGACTCCATTTTCAACGAATGCGACGAGAATTTAGAAAGCGTCTTGTCAGAAATCCGGCTTAATTTG<br>AAAACCGAGAAGTCAACGCGCCGCCGCATTTGCGCAGAGAATCTGAACCTGGAAGCTGGGATGGTGAACCGGAACA<br>GGCTAGGGAGAAAAACCCGGTTCACTTATTTGGCTCTCGCGGAGCCGTGGCCCAAAGTTTCGGGCTTTGCCAAGGTG<br>GACCTCTCCACCGGCGAGGTGAAGAAGCATATCTATGGCGAGGAGAAGTACGGCGGGGAGCCTCTGTTTCTTCCCAG<br>AGACCCCAACTCGGAGAATGAGGACGATGGATACATTTTGGCCTTCGTTCACGACGAGAAGGAGTGGAAGTCGGAGC<br>TGCAAATCGTGAACGCCATGGATTTGAAGGTGGAAGCTACAATTCAGCTGCCCTCGAGAGTTCCGTACGGTTTTCATG	NCED1(F) 5'-GGCGAGGTGAAGAAGCATATCT-3'<br>NCED1(R) 5'-GAACGAAGGCCAAAATGTATCC-3'	115
NCED5<br>(Contig_43868)	ATGACGTCAACGCCGACGACATCATCTGGGTCGACTCTCCCGACACGTTCTGCTTCCACCTCTGGAACGCGTGGGAGG<br>AGCCCGAGTCGGCCGAGGTCGTTGTGATTGGATCCTGCATGACTCCCCCCGACTCAATCTTCAACGAGTGCGATGAGA<br>ACCTCAAAAGCGTGCTGTCCGAAATCCGGCTAAATTTGAAAACCGGCGAGTCGACTCGGCGGGCGATTTTGTCTGAAT<br>CGGATGATGTGAATTTGGAGGCGGGAATGGTGAACAGGAACCGACTTGGTAGGAAAACTCGGTTCGCTTACCTCGCA<br>ATTGCCGAACCGTGGCCGAAAGTTTCGGGTTTTGCCAAAGTAGATCTTTTCACCGGGGAGGTGAAAAAGTTTTTTAC<br>GGCGAGAAGATGTACGGTGGTGAGCCGTTTTTCGTGCCGAGCACGGCCGAGGGCGCGTCGGAGGACGACGGTTATAT<br>TCTGACGTTCGTTCACGACGAGAAGAACTGGAAGTCGGAGCTTCAGATTGTTAACGCGG	NCED5(F) 5'-TGGTGAACAGGAACCGACTTG-3'<br>NCED5(R) 5'-CTTTGGCAAAACCCGAAACTT-3'	90
GA 20-oxd<br>(Contig_64541)	GTTTACCAAGAATACTGTGATGCCATGAACAACCTCTCCAATGCGATAATGGAGCTACTCGGAATGAGCCTAGGCGTTG<br>GACGAAACTATTTCAAAGAGTTCTTCGAAGGAAATGATTCGATCATGAGGTTGAACTACTACCCAATGTGCCAAAAAC<br>CAGACTTAACACTAGGAACCGGGCCTCACTGTGATCCCACATCCTTAACAATCCTTCATCAGGATCAAGTCGGCGGAC<br>TTCAAGTGTTCGTAGATGATAAATGGCACTCCGTTACTCCTAAGCTTGACGCTTTCGTCGTCAACATTGGCGATACATTT<br>ATGGCTCTATCAAATGGGATTTAC	GA(20)ox1(F) 5'-ACTACCCAATGTGCCAAAAACC-3'<br>GA(20)ox1(R) 5'-GCCGACTTGATCCTGATGAAG-3'	95
GA 20-oxd<br>(Contig_33744)	GAGAAGAATTCAAGGAATTCGGGAGGGTTTACCAAGATTATAGTGAGGCTATGAGCACACTTTCTATTGGGATCATGGA<br>ACTTCTGGGACTGAGCCTTGGAGTCGACAGAGCTTACTTCAAGGAGTTTTTCGAAGACAATGATTCGATAATGAGGCT<br>TAATTACTACCCACCATGCCAGAAACCTGAGCAGACTTTAGGCACTGGCCCTCATTGTGACCCAACTTCTTTGACCATT<br>CTTCACCAAGACCAAGTTGGAGGCCTTGAAGTCTTTGTTGATGATCAATGGCACTCCATTAGCCCTAATTTAAATGCCT	GA(20)ox2(F) 5'-TAATTACTACCCACCATGCCAGAA-3'<br>GA(20)ox2(R) 5'-TCCAACTTGGTCTTGGTGAAGAAT-3'	100

	TTGTAGTCAACATTGGTGACACCTTCATGGCTCTTTCAAACGGGAAGTACAAGAGCGGGCTGCACAGGGCAGTGGTG<br>AACAGTGAAACACCAAGGAAGTCTCTTGCATTCTTCTTGTGTCC		
GA 2-oxd<br>(Contig_5525)	ATGGTGGCTGATGGGCTGGGGATTGATCAAAAGAATGTGCTGAGCAAGCTTTTGAAAGATGAGAAGAGTGACTCATGT<br>TTCAGGCTGAACTACTATCCGCCATGCCCAGAGCTTCACGCTTTGAGTGGACGAAGTTTGATAGGGTTTGGGGAGCAC<br>ACAGACCCACAGATCATTTCTGTCCTGAAATCCAACAACACATCAGGCCTGCAGATCAGTCTCAAGGATGGGACTTGG<br>GTTACAGTCGCACCTGATCAGACTGCCTTTTTCATCAATGTTGGTGATTGCTTGCAGGTGATGACTAACGGGCGGTTTA<br>AGAGCGTGAAACATAGGGTTTTGGCTGACACACAAAGTTCAAGGGTTTCAATGATCTACTTTGGAGGGCCACCTTTGA<br>GTGAGAAGATAGCACCTCTGCCGTCTCTAATGGCAGAAGGAGAAGAAAGCTTGTACAAGGAGTTCACGTGGAGTGAA	GA(2)ox1(F) 5'-GCACACAGACCCACAGATCATTT-3'<br>GA(2)ox1(R) 5'-ACTGTAACCCAAGTCCCATCCTT-3'	90
ACO<br>(Contig_15267)	ATGACAAGGTCAGTGGCCTCCAGCTCCTCAAGGATGGTGAATGGGTGGATGTCCCCCCAATGCACCACTCCATTGTCA<br>TAAACTTAGGTGACCAGATTGAGGTGATCACCAATGGGAAGTACAAAAGTGTGATGCACCGGGTGATAGCTCAGTCGG<br>ATGGGACCAGAATGTCGATAGCCTCGTTCTACAACCCAGGCAACGACGCATTCATCAGCCCAGCACCGGCAGTGCTTG<br>AGAAGAAAACTGGGGACGCCCCAACTTATCCCAAGTTTGTGTTTGATGACTACATGAAGCTGTATTCTGGCCTGAAAT<br>TCCAAGCCAAGGAGCCAAGATTTGAAGCTATGAAGGCCAAGGAACCCACCCCTGTTGCAACTGCC	ACO(F) 5'-GGATGGGACCAGAATGTCGAT-3'<br>ACO(R) 5'-CCCCAGTTTTCTTCTCAAGCA-3'	95
ACS<br>(Contig_23001)	ATGCATTTCTTGTGCCCGTTCCTTATTATCCAGGTTTTGATCGAGATTTGGGATGGCGAACGGGAGTACAACTGATTCCG<br>GTTGCCTGTGACAGCTCCAACAATTTCAAAGTCACCAGAGCAGCATTGGAAGCTGCCTATGAGAAAGCTCAGAAGGC<br>AAACATCAGAGTAAAGGGCTTGCTCATTACTAACCCCTCAAACCCCTTAGGTACTGTCCTTGACCGAGACACCCTCATA<br>AGTCTAGTGACATTCATCAACGAAAAGAAAATCCATCTAGTCTGCGATGAAATCTATGCTGCCACCGTGTTCAGTCAGC<br>CAAGTTTCATAAGCATAGCCGAGATCATAGAGGAAAACATCGGATGCAACCGCAATCTTATTCACATTGTGTACAGTCT<br>TTCAAAAGACATGGGGTTCCCTGGCTTCAGAGTTGGCATCGTATATTCCTACAATGACGCTGTCGTTAATTGCGCACGA<br>AAGATGTCGAGTTTTGGATTGGTTTCGACACAAACTCAGCATCTGATTGCATCAATGCTGTCAGACAATGAGTTTGTGA<br>AGAGATTTATAGCACAAAGTGCTAAA	ACS(F) 5'-TCCATCTAGTCTGCGATGAAATCT-3'<br>ACS(R) 5'-GCATCCGATGTTTTCCTCTATGAT-3'	95
YUCCA4<br>(Contig_7052)	ATGGCTGCCATTGAAGCTTGTAGACAGGGTCCTATTGCTACTGGCCAATTTCATCCTAGGCAACACAGACCAATTAGGG<br>CTCAAAAGGCCCAAAACTGGCCCTATTGAGCTCAAGAATGTCACTGGGAAAACCCCAGTTCTTGATGTTGGAGCGTTG<br>TCACAAATTAAATCTGGCAAAATAAAGGTGATGGAAGGGGTGAAGGAGATAACAAGAAATGGAGCAAGATTTATGAA<br>TGGACAGGAAAAGGAGTTTGATTCTATAATCTTAGCAACTGGGTATAAAAGCAATGTGCCTACTTGGCTCAAGGGTTGT<br>GACTTTTTCACCAAAGATGGGATGCCAAAAGCACCCTTTCCCAACTGCTGGAAAGGAAGTGATGGACTCTATACAGTT<br>GGATTCACAAGAAAAGGGCTTCTTGGAACGGCTTCTGACGCTGTTGCGATAGCCAAGGATATTACTCAGCTTTGG	YUCCA4(F) 5'-GGCTCAAGGGTTGTGACTTTTT-3'<br>YUCCA4(R) 5'-AGCCCTTTTCTTGTGAATCCAA-3'	112
Cytokinin synthase<br>(Contig_25970)	TCAAGGCTCTCCATCGACCTAGCCACCTGTTTCCCGGCAGAAATCATAAACTCCGACAAAATGCAAGTCTACGAAGGC<br>CTTGACATAGTCACCAACAAAATAACCAAAGAAGAGCAACGTGGTGTACCGCACCATTTGCTAGGGATGCTAGATCCT<br>CATGAAGATTTTACTGCCAGGGATTTTTGTGACCTAACCTCAATTGCCATTGAATCCATTTTAGGCCGTGATCGGCTTCC<br>AATCATCGTTGGAGGTTCCAACTCTTACATCGAGGCGTTGATCGATGATTATGACTATAAATTTCGGTCCAAGTATGACT<br>GTTGCTTTTTATGGGTAGATGTGTCTACACCCGTTCTGCACTCTTTTGTGTCAAAACGGGTAGACCACATGGTTCACAA<br>CGGAATGGTGGATGAGGCGAGAGAGTACTTTGATCCCAATGCAGATTACACGAAAGGGATTCGAAGAGCAATAGGGG<br>TCCCTGAATTCGATAAGTACTTCAGGTATGGGCCATTT	ZTNSY(F) 5'-TTTTTGTGACCTAACCTCAATTGC-3'<br>ZTNSY(R) 5'-GATCAACGCCTCGATGTAAGAGTT-3'	100
